# Recombinant RBD-based subunit vaccines incorporating high-frequency mutation sites elicit cross-immunity and robust protection against SARS-CoV-2

**DOI:** 10.3389/fmicb.2026.1806270

**Published:** 2026-06-09

**Authors:** Zhiguang Ren, Xiawei Liu, Weiyang Sun, Wei Jiang, Yiwen Li, Xiaonan Gao, Chengyu Hu, Menghan Zhu, Yongkun Zhao, Yuanguo Li, Xianzhu Xia, Zhengyan Yang, Jing Liu, Xinrui Lv, Tiecheng Wang, Daxiang Cui

**Affiliations:** 1 Henan Intelligent Diagnosis and Treatment Engineering Technology Research Center, The First Affiliated Hospital, Henan University, Kaifeng, China; 2 Key Laboratory of Jilin Province for Zoonoses Prevention and Control, National Key Laboratory of Pathogen Microorganisms Biosafety, Academy of Military Medical Sciences, Changchun, China; 3 State Key Laboratory of Antiviral Drugs, School of Pharmacy, School of Basic Medicine Sciences, Henan University, Kaifeng, China; 4 Jiangsu Co-innovation Center for Prevention and Control of Important Animal Infectious Diseases and Zoonoses, Yangzhou University, Yangzhou, China; 5 Kaifeng Key Laboratory for Infectious Diseases and Biosafety, The First Affiliated Hospital of Henan University, Kaifeng, China

**Keywords:** high-frequency SARS-CoV-2 RBD mutation sites, RBD, recombinant subunit vaccines, SARS-CoV-2, universal vaccine

## Abstract

**Introduction:**

Severe Acute Respiratory Syndrome Coronavirus type 2 (SARS-CoV-2), a highly pathogenic coronavirus (CoV) belonging to Coronavirus B, has triggered outbreaks or pandemics. Currently, many variants of SARS-CoV-2 have evolved, which exhibit severe immune evasion and imprinting resistance to existing vaccines and cause infected individuals to develop Post-Acute Sequelae of SARS-CoV-2 (also termed Long-COVID). This underscores the public health importance of developing effective vaccines with broad-spectrum efficacy against the SARS-CoV-2 variant and other CoVs with pandemic potential.

**Results:**

In this study, we expressed three tandem-repeat dimeric recombinant RBD proteins using an insect-baculovirus expression system integrated with the high-frequency SARS-CoV-2 RBD mutation sites. We performed immunogenicity assessment and attack protection tests. The date showed that these innovative SARS-CoV-2 RBD recombinant protein vaccines could show varying degrees of cross-immunity response and potent protection against SARS-CoV-2.

**Conclusion:**

Overall, our results indicated that these recombinant RBD subunit vaccines could serves a promising platform for a universal vaccine against SARS-CoV-2 and its variants, and provide a new perspective for the design of other future pandemic vaccines.

## 1 Introduction

Since its emergence in 2019, SARS-CoV-2 has caused unprecedented global health and economic disruptions (Zhu et al., 2020). The World Health Organization (WHO) declared COVID-19 a pandemic on 11 March 2020, and as of 23 March 2025, over 778 million confirmed cases have been reported worldwide, an increase of 9,200 cases on previous 7 days.^1^ While many patients infected with the virus exhibit mild to moderate symptoms, including coughing, fever, and headache, the condition progresses to severe pneumonia, respiratory failure, and other serious complications in some patients (Lundstrom et al., 2023; Bramble et al., 2023). Therefore, developing broad-spectrum vaccines capable of neutralizing diverse SARS- CoV-2 variants and other coronaviruses with pandemic potentialremains a critical priority.

The receptor-binding domain (RBD) of SARS-CoV-2 binds to its cellular receptor angiotensin-converting enzyme 2 (ACE2) and is a key molecule for the efficient entry of the virus into the host cell (Saito et al., 2022; Xia et al., 2022; Wang et al., 2023; Lin et al., 2023; Wu et al., 2022). However, the RBD of SARS-CoV-2 has been found to mutate rapidly and is an important site for distinguishing between variants, with at least six variants of interest (Alpha, Beta, Gamma, Delta, Lambda, and Omicron) having emerged to date (Simeoni et al., 2021; Ivanov et al., 2024; Thoms et al., 2020). High-frequency mutations in RBD not only enhance the infectivity and transmissibility of these variants but also weaken the protection provided by vaccines or humoral immunity induced by previous natural infections (i.e., enhanced immune escape) (Dickey et al., 2023; Li et al., 2024). Currently, available SARS-CoV-2 vaccines are relatively ineffective against the emerging Omicron subvariant, which significantly evades neutralizing immunity or protective efficacy against wild-type (WT) strains or earlier variants (Yuan et al., 2020; Frolov et al., 2023). To date, some variants have demonstrated changes in clinically relevant characteristics, including disease severity, immune escape, and susceptibility to treatment. Studies have shown that the immune epitopes of RBD are capable of inducing neutralizing antibodies and T-cell immune responses, making it an ideal target as a means of developing effective vaccines (Anzai et al., 2024; Zhang et al., 2024). Several studies have shown that vaccines with RBD as antigen show good protection in both animal models and human trials (Liu et al., 2024; Zhou et al., 2024; Yang et al., 2021). The vaccines that integrating hotspot mutations leading to immune escape into the RBD could broaden the activity spectrum of antigen-induced antibodies (An et al., 2024; Liang et al., 2025). However, the low molecular weight of RBD elicits limited immune responses when used individually, so it is often necessary to design RBD as multimers of multivalent antigens to achieve the desired immunogenic effect (Yang et al., 2021; Chao et al., 2024; Gao et al., 2023; Liu et al., 2020). In conclusion, the strategy of using multimeric RBD recombinant proteins containing mutation sites as vaccines has become a direction of research for the next generation of broad-spectrum coronavirus vaccines.

AddaVax™ is a squalene-based water-in-oil nanoemulsion, with a formulation similar to MF59®, where squalene is an oil that is more easily metabolized than the mineral oil used in Freund’s adjuvant (Kang et al., 2021). Compared to aluminum adjuvants, it enhances the uptake and presentation efficiency of antigens, further increasing the entry of antigen-presenting cells into the lymph nodes, which can better activate T cells and B cells to generate an immune response (Ahuja et al., 2024).

In this study, we selected five RBD mutation sites: K417/L452/T478/E484/N501. They are present in many lineages, represent the most impactful sites associated with immune evasion and viral entry, and are the key immunodominant sites that are recurrently targeted by neutralizing antibodies (Li et al., 2024; Zhang et al., 2023; Randriantseheno et al., 2024). We constructed three tandem RBD-dimers: NNR (RBD1 + RBD1, with K417N/L452R/T478K/E484K/N501Y mutation sites), TTR (RBD2 + RBD2, with K417T/L452R/T478K/E484K/N501Y mutation sites), and NTR (RBD1 + RBD2, combining both mutation sets). The recombinant RBD proteins were expressed using an insect-baculovirus expression system and combined with the adjuvant AddaVax for intramuscular immunization of mice and golden gophers to evaluate the immunogenicity of the recombinant protein vaccine and its protective effect against SARS-CoV-2 BA.2 infection. The vaccine was tested have the potential of inducing cross-immunity protection against different SARS-CoV-2 subtypes, providing a basis for the development of a new coronavirus vaccine with universal protective activity.

## 2 Materials and methods

### 2.1 Facilities and ethics statement

All Animals were treated in accordance with Chinese welfare and ethical guidelines for laboratory animals. The protocol was approved by the Animal Welfare and Ethics Committee. All highly pathogenic virus experiments were performed in a Biosafety Level 3 (BSL3) facility.

### 2.2 Viruses, cells, antibodies, and animals

SARS-CoV-2 (IME-BJ05-2020, GenBank Accession No. MT291835) and SARS-CoV-2 BA.2 (hCoV-19/ Jilin/JSY-CC5/2022, GISAID Accession No. EPI_ISL_18435548) are kept in the laboratory of Key Laboratory of Jilin Province for Zoonoses Prevention and Control, National Key Laboratory of Pathogen Microorganisms Biosafety, Academy of Military Medical Sciences. Insect baculovirus system dual expression vector pFastBac1, *Spodoptera frugiperda* (Sf9) cells, insect cell High Five suspension cells, and Vero E6 cells were prepared and preserved in the laboratory of Key Laboratory of Jilin Province for Zoonoses Prevention and Control, National Key Laboratory of Pathogen Microorganisms Biosafety, Academy of Military Medical Sciences. Rabbit anti-SARS-CoV-2 (COVID-19) Spike RBD antibody (GTX135385) was purchased from GeneTex, Goat Anti-Rabbit IgG H&L (FITC) (ab6717) and HRP-conjugated Goat Anti-Rabbit IgG (ab6721) were purchased from Abcam. Young female BALB/c mice aged 6–8 weeks, and elderly female BALB/c mice aged 6–8 months and female golden gophers aged 4–6 weeks were purchased from Beijing Viton Lever Laboratory Animal Technology Co. The animals were kept under specific pathogen-free conditions, with ad libitum access to food and water. Before immunization or virus challenge, animals are acclimated to the environment for 7 days, and monitored twice daily.

### 2.3 The generation of tandem RBD expression vectors

A new RBD protein containing five mutation sites was constructed based on the reference genome sequence of the wild-type novel coronavirus Spike RBD (NC_045512.2). The two RBDs were linked using flexible peptides (GGGGS) and then connected to the pfastbac dual shuttle vector. A bee venom signal peptide was added to the N-terminus of the recombinant protein to promote its secretion expression in insect cells, and a his tag was added to the C-terminus to assist in the purification of the recombinant protein.

### 2.4 Indirect immunofluorescence assay

Sf9 cells were infected with three recombinant RBD baculoviruses at MOI 2. Cells were fixed with 4% paraformaldehyde fixative 48 h after infection. The cells were then sequentially incubated with Rabbit anti-SARS-CoV-2 (COVID-19) Spike RBD antibody (1:500) for 3 h at room temperature, and Goat Anti-Rabbit IgG H&L (FITC) (1:500) was incubated for 1 h at room temperature.

### 2.5 Protein preparation, SDS-PAGE identification, and Western blot analysis

P3 generation recombinant baculovirus with MOI of 2 cultured in adherent Sf9 cells was inoculated into High Five suspension cells and cultured for 72 h. After centrifugation at 4 °C and 5,000 rpm, the collected cell supernatant was filtered through a 0.45 μm membrane and combined with a nickel gravity column for purification. The protein eluate was collected and dialyzed through an ultrafiltration centrifuge tube and PBS to obtain high purity and high concentrations of recombinant protein. Protein concentration was determined using the BCA Protein Assay Kit (Beyotime) according to the instructions. Samples were prepared with sodium dodecyl sulfate (SDS) and placed in boiling water for 10 min. Samples (20 μg) were separated by 10% SDS-PAGE and then transferred to a polyvinylidene difluoride (PVDF) membrane. Rabbit anti-SARS-CoV-2 (2019-nCoV) Spike RBD antibody (1:200) was used as primary antibodie and HRP-conjugated Goat Anti-Rabbit IgG (1:5000) was used as secondary antibodies. Finally, the image was captured using an enhanced chemiluminescence detection kit (Beyotime) with a Tanon-5200 Imaging System (Tanon, China).

### 2.6 Animal immunization and viral challenge

One hundred and twenty 6–8 weeks female BALB/c mice were randomly divided into 8 groups of 15 animals each, 60 healthy 6–8-month-old female BALB/c mice were randomly divided into 5 groups of 12 animals each, and 40 4–6 week old golden gophers were randomly divided into 4 groups of 10 animals each. The experimental animals were immunized with 10 μg recombinant protein (Among others, 6–8 weeks female BALB/c mice were immunized with 1 μg recombinant protein at the same time) by intramuscular injection combined with adjuvant AddaVax (Invivogen) at a 1:1 ratio on days 0 and 21, respectively. BALB/c mice of 6–8 months of age on day 14 after immunization and golden gophers and control animals (PBS and AddaVax groups) on day 35 after immunization were transferred to a BSL-3 laboratory. Each mouse was lightly anesthetized with isoflurane and then infected with 50 μL of 10^5^ TCID50 SARS-CoV-2 BA.2 live virus by nasal drip (25 μL per nostril) for the viral challenge protection experiments.

### 2.7 Elisa

Orbital whole blood was collected from five mice in each group at 0, 3, and 5 weeks after immunization, and serum was collected and stored at −20 °C. SARS-CoV-2(2019-nCoV) Spike RBD or SARS-CoV-2 BA.2 Spike RBD proteins (Beijing Yibiao Shenzhou Co., Ltd., China, 0.2 μg per well) were coated on a 96-well microtiter plate (Costar, United States), and then incubated at 4 °C overnight. After washing the plate, the wells were blocked with 1% BSA at 37 °C for 2 h. The blocking solution was then discarded. Serum samples were diluted 200-fold with 0.5% BSA and added to 96-well plates incubated at 37 °C for 1.5 h as previously reported (Li et al., 2024; Zhang et al., 2023). After another round of washing, the antibodies IgG, IgG1, and IgG2a diluted with 1% BSA were added and incubated at 37 °C for 1 h. After washing, a TMB termination solution was added. After color development, the OD450 nm value was measured using a spectrophotometer. The results were expressed as the positivity index (ratio of OD450 of the experimental group to that of the PBS control group) (Zhang et al., 2023; Randriantseheno et al., 2024).

### 2.8 Neutralization assay

The mouse serum was inactivated at 56 °C for 30 min; the serum was diluted in a biosafety cabinet with opti-MEM containing 1% penicillin–streptomycin in a 96-well plate, starting at 1:20 and diluting at a 2-fold ratio. The diluted serum was incubated with 100 TCID50 of SARS-CoV-2 (2019-nCoV) for 1 h at 37 °C. Upon completion of incubation, the serum-virus mixture was added to Vero E6 cells prepared in advance and incubated at 37 °C for 72 h. A PBS-negative cell control group and a blank cell control group were set up in the cells. After incubation, the cells in each well of the 96-well plate were observed under an electron microscope, and the neutralization potency of the immunized mouse sera was calculated according to the Reed-Menuch method (the known magnitude of neutralization titers protective thresholds is 1:80). The neutralization assay were conducted at the Biosafety Level 3 Laboratory (BSL-3).

### 2.9 ELISpot

A total of 5 × 10^5^ mouse isolated splenic lymphocytes were inoculated onto ELISpot plates (Mabtech, Sweden) and and stimulated with SARS-CoV-2 (2019-nCoV) Spike RBD protein or SARS-CoV-2 BA.2 Spike RBD protein (10 μg/well). After washing the ELISpot plates 5 times with PBST, the cells were incubated with biotin-labeled BVD6-24G2 and R4-6A2 antibodies (1:1000) provided in the ELISpot kit for 2 h at room temperature. The plates were then washed again and incubated with streptavidin-conjugated ALP (1:1000) for 1 h at room temperature. After the addition of BCIP/NBT substrate, spot-forming cells (SFC) in the plate were counted with an ELISpot reader (Multispotreader Spectrum, Germany).

### 2.10 Splenocyte supernatant multi-factor assay

The prepared mouse splenocyte suspension was added into 24-well plates at 500 μL/well, and SARS-CoV-2(2019-nCoV) Spike RBD protein or SARS-CoV-2 BA.2 Spike RBD protein was added as a stimulant (final concentration of 5 ng/μL, three replicate wells were set up for one mouse at the same time). The plates were incubated in a cell culture incubator at 37 °C for 60–72 h. The supernatant of cultured splenocytes was collected and stored at −80 °C, and then sent to Leitz Biotechnology Shanghai for Luminex assay using Th1/Th2 Cytokine 11-Plex Mouse Panel Multifactor Kit.

### 2.11 Flow cytometry

Detection of T cell activation was further assessed by flow cytometry. On the 10th day after booster immunization, spleens and lung lymph nodes from mice were collected, and single-cell suspensions were prepared as previously described (Edelson et al., 2010). Collect the spleen and gently grind it in complete RPMI 1640 containing 10% heat-inactivated FCS (Gibco), 50 mg/mL streptomycin and 50 U/mL penicillin (Solarbio, Beijing, China). To isolate lymphocytes from lung sections, digest the lung tissue in complete RPMI 1640 with 250–300 U/mL type IV collagenase (Sigma) at 37 °C for 30 min, then filter through a 70 μm filter. Then resuspend the cells from the spleen and lung in red blood cell lysis buffer (Solarbio, Beijing, China) at room temperature for 2 min. After washing the cells twice with RPMI medium, dilute them to the desired concentration. A total of 4 × 10^6^ cells per well were then seeded into 6-well U-bottom plates, followed by stimulation with SARS-CoV-2(2019-nCoV) Spike RBD protein or SARS-CoV-2 BA.2 Spike RBD protein (20 μg/well) for 2–16 h. Cells were stained using the Zombie NIR™ Fixable Viability Kit (Cat. No.: 423106), set up blank control and live/dead dye single-staining tubes. Spleen cells were incubated at 4 °C for 30 min with a mixture of CD45 (APC), CD3 (BV510), CD4 (FITC), CD8 (BV785), IFN-γ (BV421), IL-2 (BV605), and TNF-α (PE-Cy7) antibodies, or lung cells were incubated at 4 °C for 30 min with a mixture of CD45 (APC), CD3 (BV510), CD4 (FITC), CD8 (BV785), CD69 (BV421), and CD103 (PE-H) antibodies. Also set up spleen /lung single-staining antibody control tubes and spleen /lung mixed-staining antibody tubes. Flow cytometry analysis was performed on an LSRFortessa flow cytometer (BD Biosciences, United States). Data were analyzed using FlowJo V.10.8.1 (Tree Star).

### 2.12 Histology

The lungs of 3 mice in each group were collected and stained for histopathological qualitative analysis. Briefly, the lungs were fixed in 4% paraformaldehyde solution for 2 weeks, and cut into 3 μm sections after embedded with paraffin. Hematoxylin and Eosin (H&E) stain was used to observe histopathological changes. Immunohistochemistry (IHC) analysis using an anti-RBD protein antibody (GeneTex, GTX135385) at a dilution of 1:50 was conducted to evaluate the distribution of SARS-CoV-2 within lung tissues. For H&E, lung tissue damage was semi-quantitatively scored based on the proportion of affected alveolar area, inflammatory cell infiltration, and bronchial injury. A scoring system ranging from 0 to 4 was used, with higher scores indicating more severe pathological changes. For IHC, the number of SARS-CoV-2 antigen-positive cells was quantified and expressed as positive cells per mm^2^ in representative lung fields.

### 2.13 Statistical analysis

Data were analyzed using GraphPad Prism 6.0. Group comparisons were made by two-way or one-way ANOVA with Dunnett’s *post hoc* test. Results represent independent biological replicates (mean ± SD). For the technical replicate measurements corresponding to a single biological replicate, the mean value was taken for statistical analysis and graphing. Error bars represent the standard deviation (SD) of each group (*p < 0.05; **p < 0.01; ***p < 0.001; ****p < 0.0001; ns, not significant).

## 3 Results

### 3.1 Construction, preparation, and characterization of recombinant subunit vaccines NNR, TTR, and NTR

**Figure 1:**
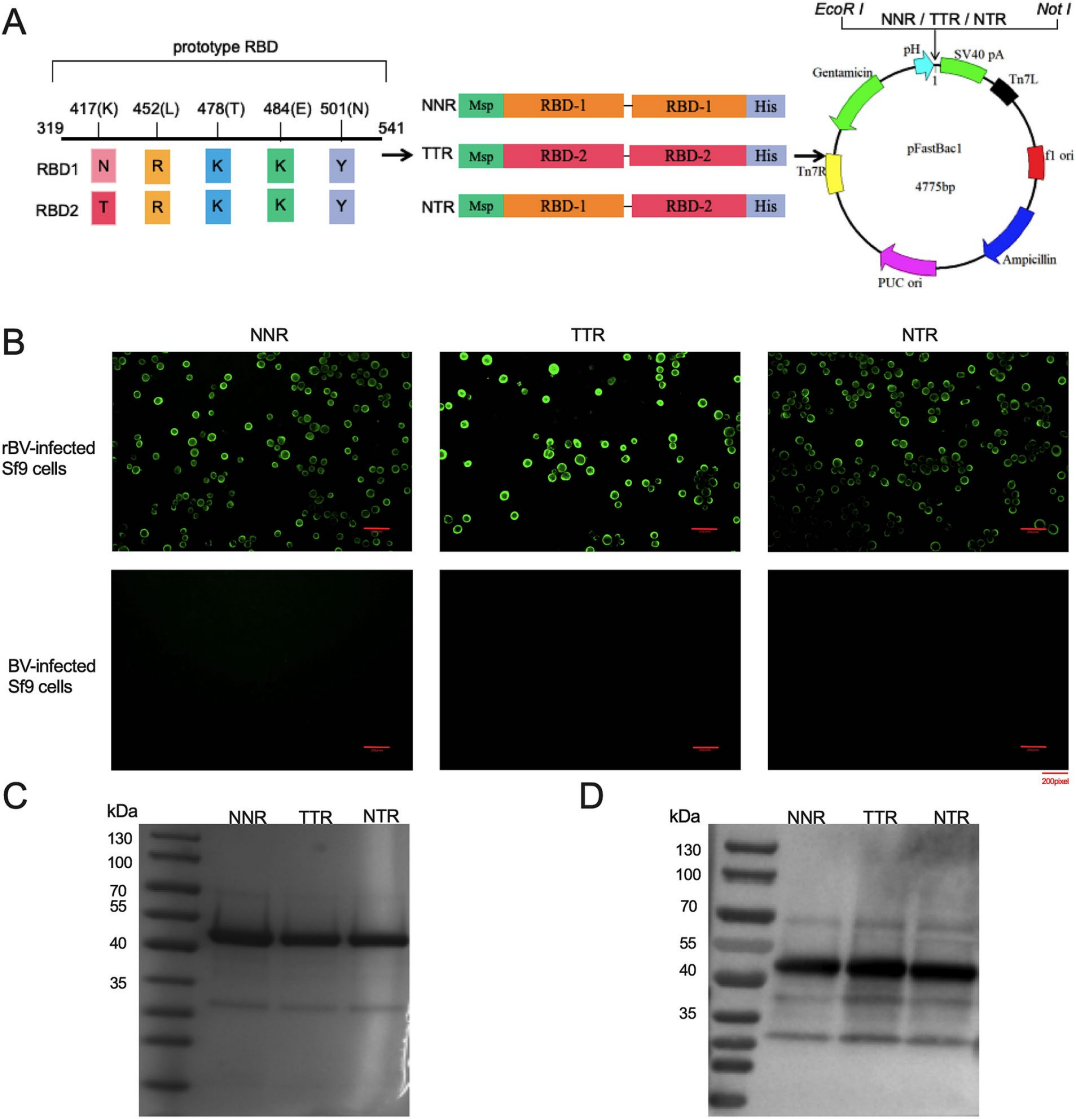
Construction and identification of SARS-CoV-2 RBD recombinant subunit vaccines. **(A)** Schematic representation of the construction of recombinant subunit vaccines of NNR, TTR, and NTR. The first one indicating the mutation sites, the second one indicating the genes construction, and the third one indicating the expression plasmids construction. Msp: signal peptide, RBD-1: containing five variable sites K417N, L452R, T478K, E484K, N501Y; RBD-2: containing five variable sites K417T, L452R, T478K, E484K, N501Y; His: histidine tags; **(B)** IF detection of the expression of recombinant baculovirus rBV-NNR, rBV-TTR, rBV-NTR in Sf9 cells, and cells were infected with empty baculovirus as mock; compared to the control group, strong specific green fluorescence signals were observed in the cells infected with three types of recombinant baculoviruses: NNR-rBV, TTR-rBV, and NTR-rBV. Scale bar: 200 pixel; SDS-PAGE **(C)** and Western blot analysis **(D)** of NNR, TTR, and NTR.

The schematic diagram of constructs for NNR, TTR, and NTR is shown in Figure 1A. The Figure 1A has three parts: the first one indicating the mutations, the second one indicating the construction and the third one indicating the plasmid cloning. First, we constructed two novel recombinant proteins, RBD1 and RBD2. The RBD1 protein contains five mutation sites (K417N, L452R, T478K, E484K, and N501Y), while the RBD2 protein contains five mutation sites (K417T, L452R, T478K, E484K, and N501Y). Next, we linked any two RBD sequences using a flexible (Gly-Gly-Ser)₂ linker (GGSGGS, 6 amino acids) to construct three combinations of recombinant subunit vaccines: NNR (RBD1 + RBD1), TTR (RBD2 + RBD2), and NTR (RBD1 + RBD2). These three combinations differ specifically at the K417 amino acid mutation (N for RBD1 vs. T for RBD2). Finally, the three genes (NNR, TTR, and NTR) were inserted into the insect baculovirus expression vector pFastBac1 using the restriction enzyme cutting sites EcoRI and NotI for subsequent protein expression (Figure 1A). Indirect immunofluorescence assay (IIFA) detected the expression of RBD protein in Sf9 cells infected with the three recombinant baculoviruses, NNR-rBV, TTR-rBV, and NTR-rBV; Compared to the control group, strong specific green fluorescence signals were observed in the cells infected with three types of recombinant baculoviruses: NNR-rBV, TTR-rBV, and NTR-rBV (Figure 1B). At the same time, SDS-PAGE (Figure 1C) and Western Blot (WB) (Figure 1D) detection confirmed that the band size of the RBD protein in these three recombinant subunit vaccines was consistent with the expected 54.3 KDa (which includes the two RBD domains, the peptide linker, and the His-tag). These findings collectively confirm the successful preparation of the three recombinant subunit vaccines: NNR, TTR, and NTR.

### 3.2 Recombinant subunit vaccines NNR, TTR, and NTR elicit robust germinal center response against the SARS-CoV-2 in BALB/c mice

**Figure 2:**
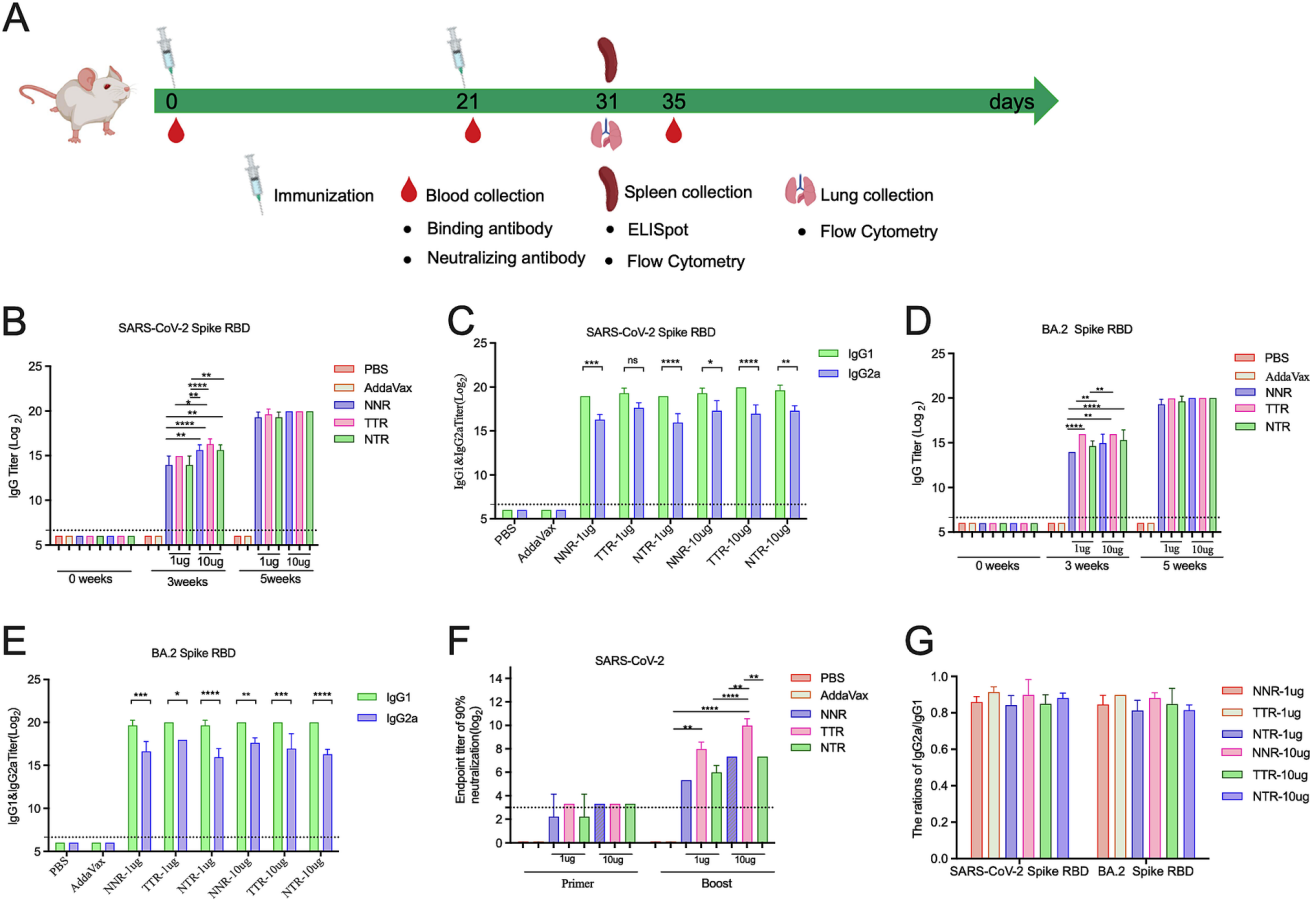
Specific serum antibodies and neutralizing antibody assays at 0, 3, and 5 weeks after immunization of young BALB/c mice with NNR, TTR, and NTR. **(A)** Schematic illustration of the immunization strategy in mice. Serum-specific total IgG antibody levels **(B)** and specific IgG antibody subtype levels **(C)** were measured in mice (SARS-CoV-2(2019-nCoV) Spike RBD protein as reactive antigen). Serum-specific total IgG antibody levels **(D)** and specific IgG antibody subtype levels **(E)** in mice (SARS-CoV-2 BA.2 Spike RBD protein as reactive antigen); **(F)** Neutralizing activity against SARS-CoV-2 of mice serum, when immunized with three different doses of recombinant proteins; **(G)** IgG2a/IgG1 ratios of mouse sera under different antigenic stimuli. The dashed lines setting in figures are that the data of the experimental group above this line is meaningful. *n* = 3 mice/group/time point. Data are shown as the means ± SD and were analyzed by one-way ANOVA (*p < 0.05, **p < 0.01, ***p < 0.001, ****p < 0.0001).

Humoral immunity is crucial for defense against SARS-CoV-2, as neutralizing antibodies can effectively block viral infection and disease progression. Here, 6–8 weeks-young female BALB/c mice were immunized intramuscularly with different recombinant protein components (combined with AddaVax as an adjuvant) at different doses, twice, 21 days apart, to evaluate the specific humoral immune responses elicited by three recombinant subunit vaccines (Figure 2A). Serum samples were collected at specific time points (0 days, 21 days, and 35 days) after immunization to test antibody titers.

First, the levels of antigen-binding IgG antibodies in the serum were tested by indirect enzyme-linked immunosorbent assay (ELISA). The results showed that the recombinant subunit vaccines induced the production of IgG antibodies against SARS-CoV-2(2019-nCoV) Spike RBD protein and SARS-CoV-2 BA.2 Spike RBD protein in mice, with the IgG antibody potency in the booster immunization group significantly higher than that in the primary immunization group, and dose-dependent in the primary immunization. In the high-dose group after booster immunization, serum antibody levels could reach up to 1:1024000. Notably, among the three recombinant subunit vaccines, TTR showed higher levels of specific antibodies during primary immunization (Figures 2B,D).

Then, the levels of neutralizing antibodies in the serum were tested by neutralization assay at the BSL-3 facility, showing no difference between the primary immunization group and the control group. After booster immunization, there were significant differences between the high immunization dose TTR group and other groups, with neutralizing antibody levels reaching up to 1:1280; there were significant differences between the low immunization dose TTR group and the NNR group, with neutralizing antibody levels reaching 1:320 (Figure 2F).

Subsequently, we evaluated the specific IgG subtype antibodies IgG1 (representative of a possible biased Th2-type cellular immune response) and IgG2a (representative of a possible biased Th1-type cellular immune response) induced by the three recombinant subunit vaccines (Jangra et al., 2022; Stevens et al., 1988). The results showed that all three recombinant subunit vaccines significantly increased the titers of IgG1 and IgG2a antibodies against SARS-CoV-2(2019-nCoV) Spike RBD protein and SARS-CoV-2 BA.2 Spike RBD protein, with IgG1 levels higher than IgG2a (Figures 2C,E). We calculated the IgG2a/IgG1 ratio for each mouse (Figure 2G) and performed a one-sample *t*-test against a hypothetical value of 1. The ratios in all vaccinated groups were less than 1 (Figure 2G), and most were significantly lower than 1 (*p* < 0.05) (date no show). The ratios of IgG2a to IgG1 revealed the vaccines can effectively induce Th1-type and Th2-type immune responses in BALB/c mice, with a predominant Th2-biased immune response (Figure 2G).

The above results indicate that the three recombinant subunit vaccines are favorable for inducing increased cross-immunogenicity, and eliciting high levels of partial cross-neutralization between ancestral strain and BA.2.

### 3.3 Recombinant subunit vaccines NNR, TTR, and NTR can promote the activation of splenic T lymphocytes and lung tissue-resident memory T (TRM) cells

On the 10th day after booster immunization with NNR, TTR, and NTR, splenocytes and lung cells were collected from 6 to 8 week-old BALB/c mice and stimulated with SARS-CoV-2(2019-nCoV) RBD protein or SARS-CoV-2 BA.2 RBD protein.

**Figure 3:**
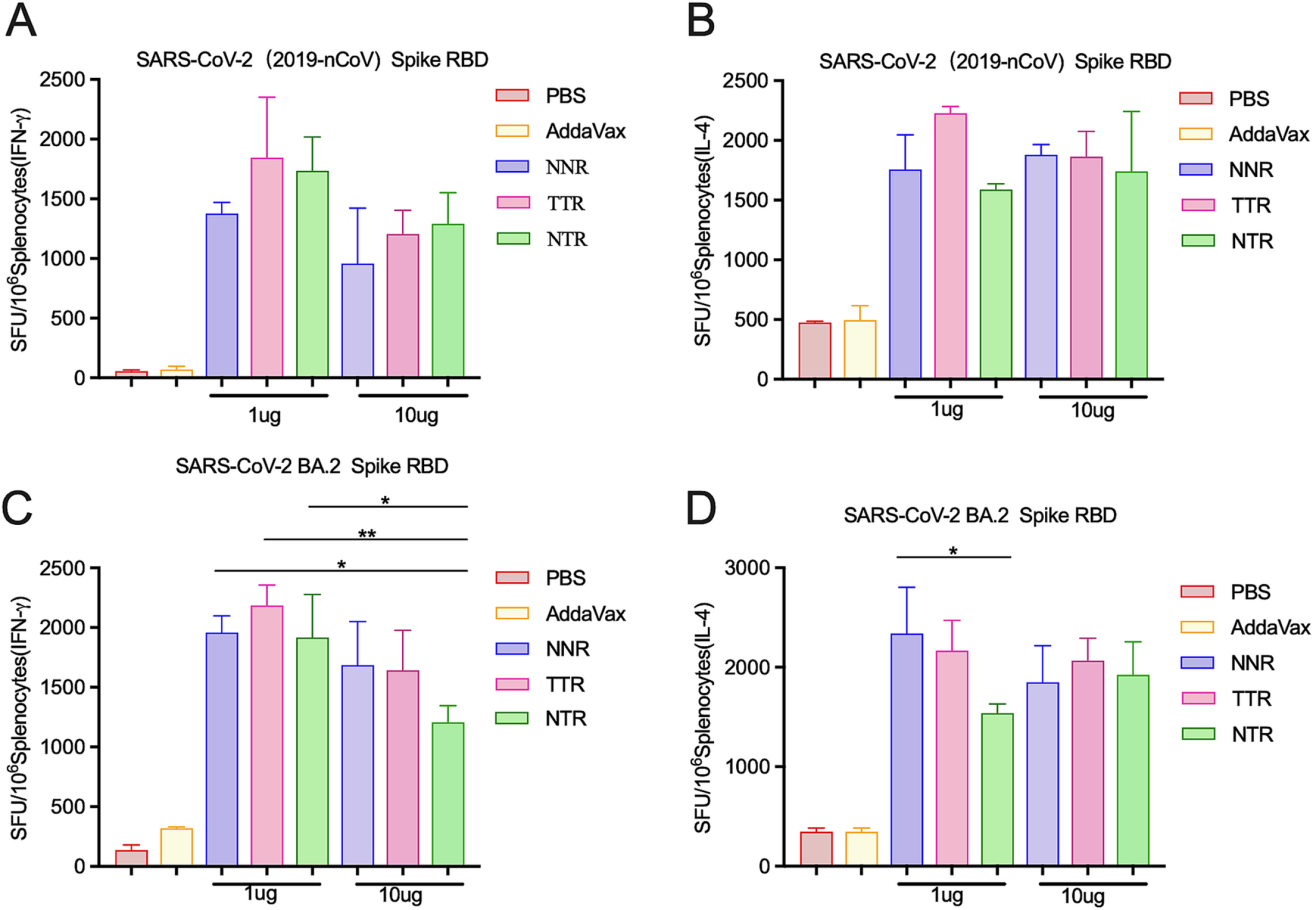
Statistical plots of the ELISPOT results: Splenocytes were isolated from immunized mice at 10 days after booster immunization at 3 weeks. The ELISpot method was used to detect the levels of IFN-γ and IL-4 secreted by splenocytes. **(A,B)** SARS-CoV-2(2019-nCoV) spike RBD protein as a stimulant, **(C,D)** SARS-CoV-2 BA.2 spike RBD protein as a stimulant. *n* = 3 mice/group/time point. Data are shown as the means ± SD and were analyzed by one-way ANOVA (*p < 0.05, **p < 0.01, ****p < 0.0001).

First, we performed enzyme-linked immunospot (ELISpot) assays to detect the secretion levels of INF-γ (produced by Th1 cells, mainly involved in cellular immunity) and IL-4 (produced by Th2 cells, mainly involved in humoral immunity) (Figure 3). This method can effectively distinguish activated T cell subsets to study the cellular immune responses induced by recombinant subunit vaccines. The results showed that the numbers of INF-γ and IL-4 spot secreted by splenocytes from mice immunized with NNR, TTR, and NTR were significantly higher than those of the control group; however, the low-dose group had the higher numbers of INF-γ and IL-4 spot than high-dose group, indicating that 1 μg of recombinant subunit vaccine was sufficient to induce high levels of T cell immune responses in mice; the numbers of IL-4 spot secreted by splenocytes from mice immunized with these three recombinant subunit vaccines were all higher than the numbers of IFN-γ spot, suggesting that the immunization mainly induced humoral immune responses, which is consistent with the results of specific antibody IgG subtype detection.

**Figure 4:**
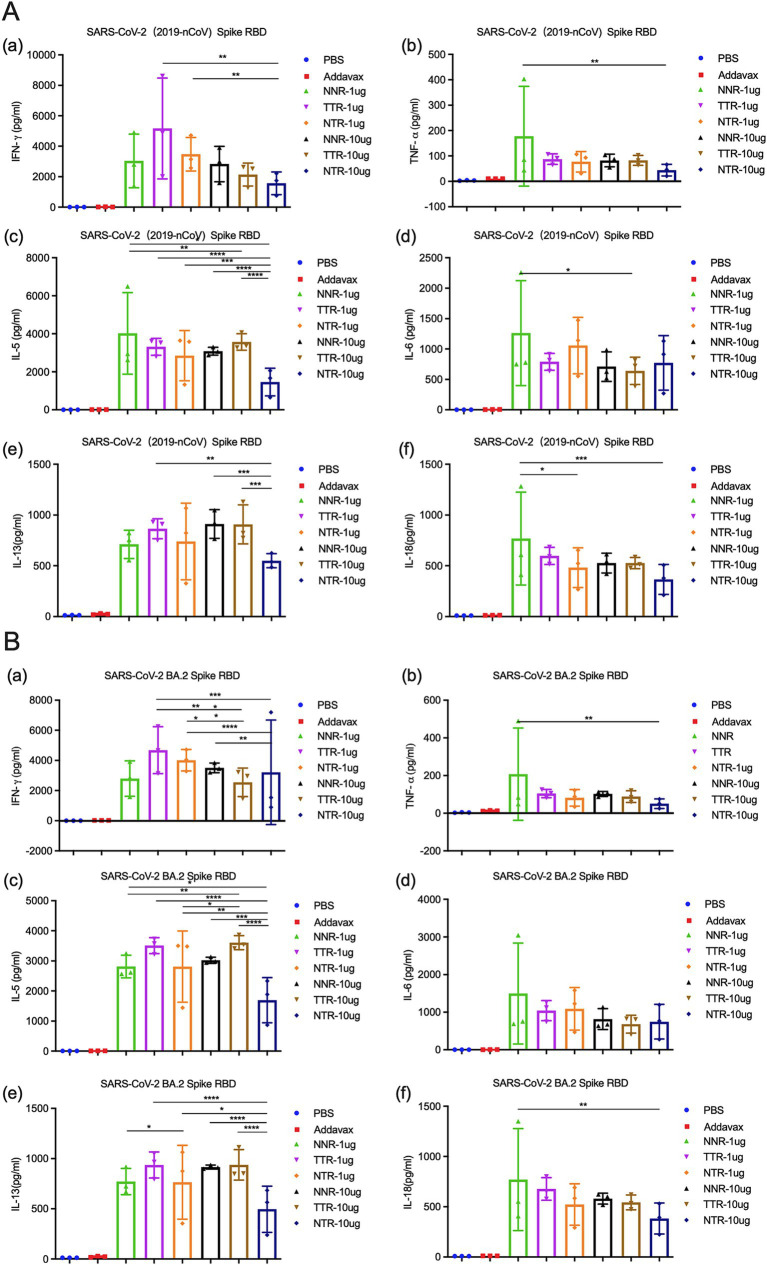
Detection results of multiparameter assays for splenocytes supernatants: Splenocytes were isolated from immunized mice at 10 days after booster immunization at 3 weeks. The Luminex was used to detect the levels of IFN-γ (a), TNF-ɑ (b), IL-13 (c), IL-5 (d), IL-6 (e), and IL-18 (f) after stimulation of splenocytes with SARS-CoV-2 (2019-nCoV) Spike RBD protein **(A)** and SARS-CoV-2 BA.2 Spike RBD protein **(B)**. *n* = 3 mice/group/time point. Data are shown as the means ± SD and were analyzed by one-way ANOVA (*p < 0.05, **p < 0.01, ***p < 0.001, ****p < 0.0001).

**Figure 5:**
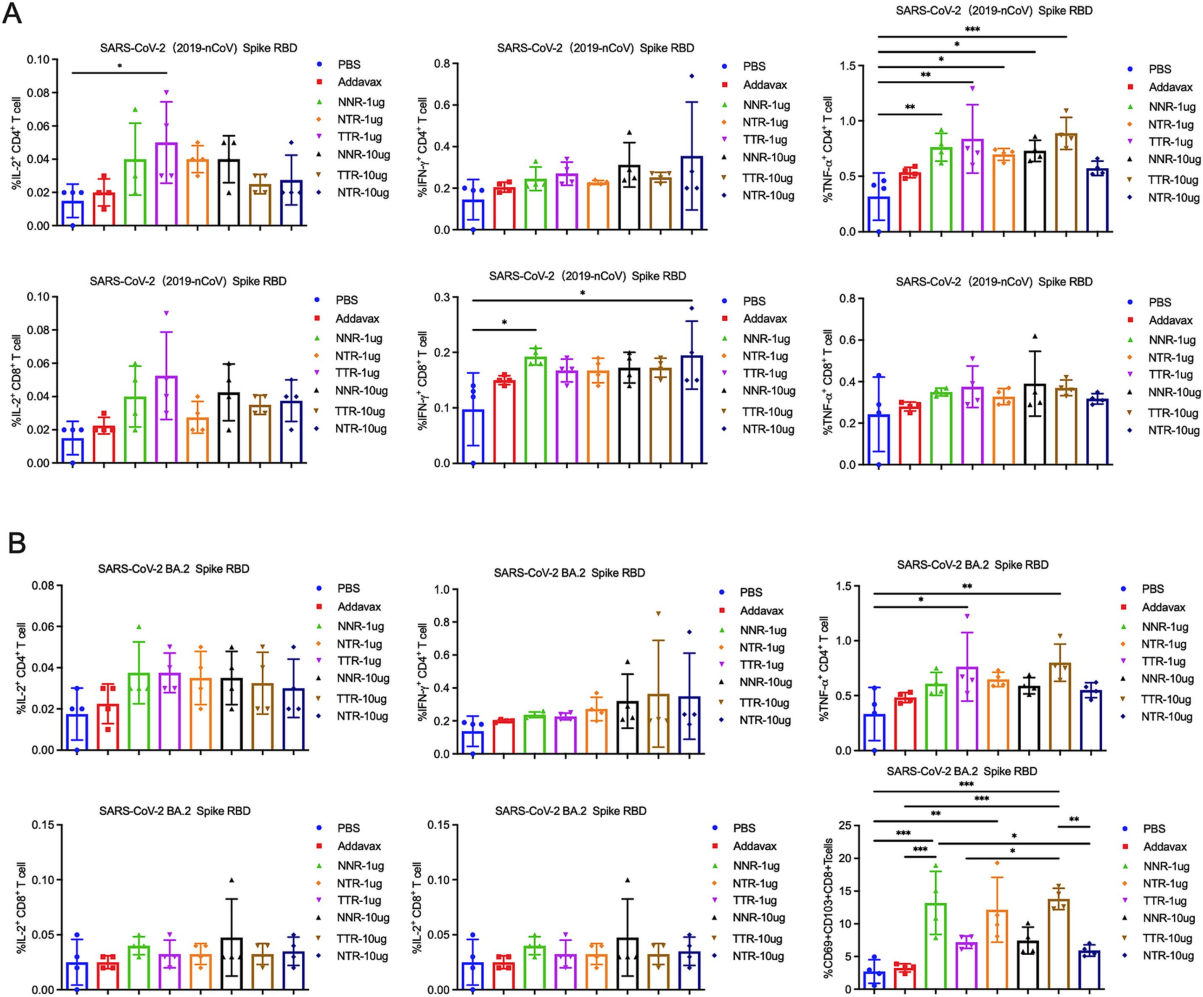
NNR, TTR, and NTR enhance intracellular cytokine secretion after immunization of BALB/c mice: Splenocytes were isolated from immunized mice at 10 days after booster immunization at 3 weeks. The Luminex was used to detect the levels of IL2^+^, IFN-γ, and TNF-ɑ secreted by CD4^+^ T cells or CD8^+^T cells after stimulation of splenocytes with SARS-CoV-2(2019-nCoV) Spike RBD protein **(A)** and SARS-CoV-2 BA.2 Spike RBD protein **(B)**. *n* = 4 mice/group/time point. Data are shown as the means ± SD and were analyzed by one-way ANOVA (*p < 0.05, **p < 0.01, ***p < 0.001, ****p < 0.0001).

Similarly, the TTR group showed the highest numbers of IFN-γ and IL-4 spot in different dose groups after stimulation with different proteins, indicating that compared to NNR and NTR, the TTR group could induce stronger cytokine production in splenocytes, thereby triggering stronger immune protection. There were no significant differences among the NNR, TTR, and NTR immunized groups in Figures 3A,B. To further verify the secretion of splenic T cell cytokines, Luminex was used to detect the levels of cytokines (IFN-γ, TNF-α, IL-5, IL-6, IL-13, and IL-18) associated with the activation of innate immunity against respiratory viruses. As expected, the results showed that the levels of cytokines produced by splenocytes from mice immunized with the three recombinant subunit vaccines were significantly higher than those of the control group after stimulation with different proteins (Figure 4). These data indicate that the recombinant subunit vaccines NNR, TTR, and NTR enhanced the secretion of splenic cell lymphokines after immunization in mice. Finally, the isolated splenocytes and lung cells were stained and analyzed by flow cytometry. Splenic lymphocytes are mainly divided into CD4^+^T cells and CD8^+^T cells. Compared to the control group, they secreted elevated levels of cytokines such as IL-2, IFN-γ, and TNF-α to varying degrees under stimulation with different proteins (Figure 5).

**Figure 6:**
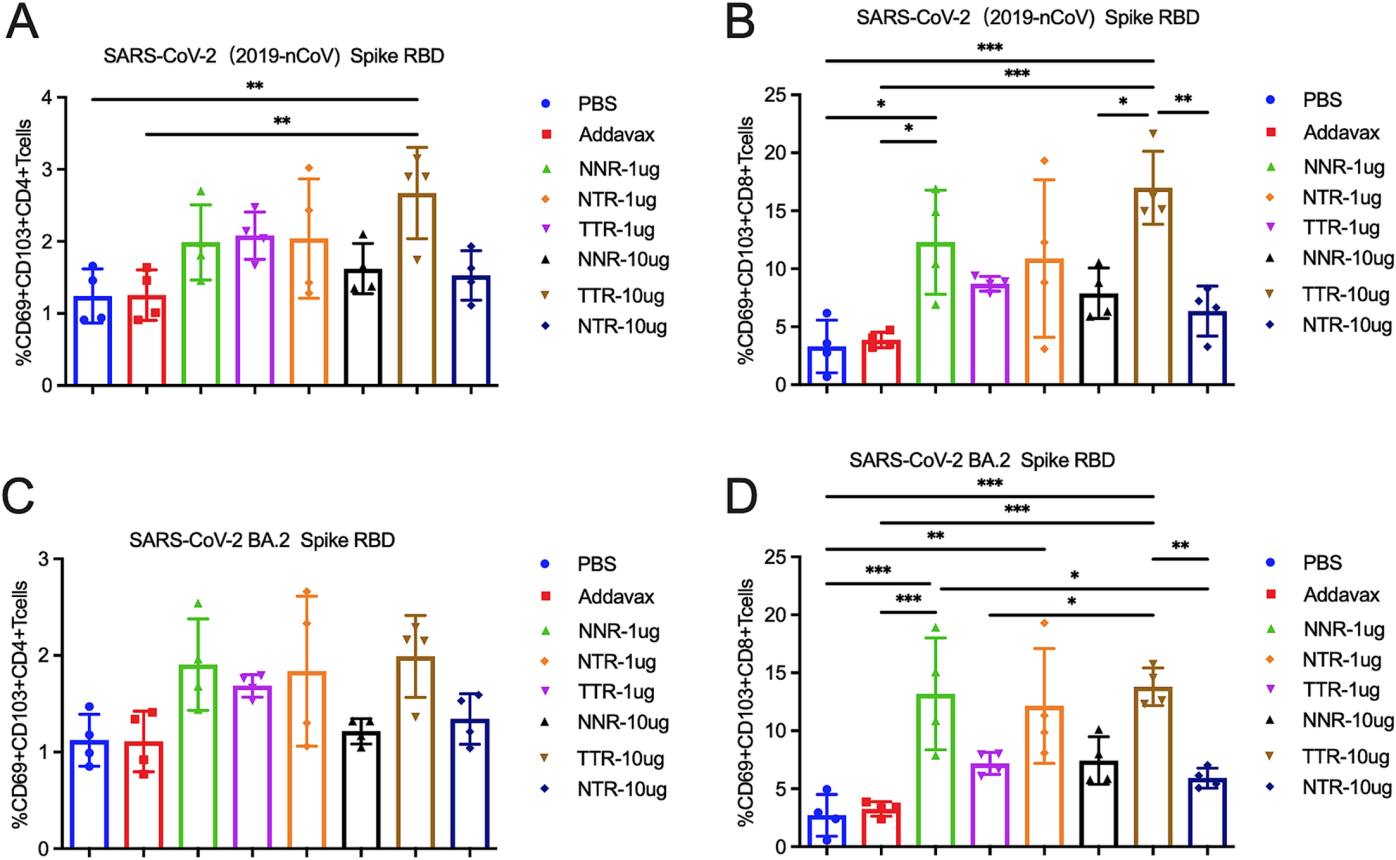
NNR, TTR, and NTR enhance lung tissue-resident memory T (T_RM_) cells cytokine secretion after immunization of BALB/c mice: Lung lymphocytes were isolated from the immunized mice at 10 days after booster immunization at 3 weeks. The flow cytometry was used to detect the levels of CD69^+^CD103^−^CD4^+^ T cells **(A)** and CD69^+^CD103^+^CD8^+^ T cells **(B)** produced lung cells after stimulation with SARS-CoV-2(2019-nCoV) spike RBD protein, and the levels of CD69^+^CD103^+^CD8^+^ T cells **(C)** and CD69^+^CD103^+^CD8^+^ T cells **(D)** produced lung cells after stimulation with SARS-CoV-2 BA.2 spike RBD protein. *n* = 4 mice/group/time point. Data are shown as the means ± SD and were analyzed by one-way ANOVA (*p < 0.05, **p < 0.01, ***p < 0.001).

Tissue-resident memory T cells (TRM cells), a type of memory T cells generated after viral infection of host cells, can stimulate the host to produce various immune responses and protective effects (Alexovič et al., 2024; Viode et al., 2024). They highly express CD69 and CD103 and do not participate in systemic circulation (Beumer-Chuwonpad et al., 2024). Flow cytometry analysis of CD69^+^CD103^−^CD4^+^ T cells and CD69^+^CD103^+^CD8^+^ T cells in lung cells showed that compared to the control group, the levels of CD4^+^ T cells and CD8^+^ T cells in lung cells were increased after stimulation with different proteins (Figure 6).

Therefore, the above results indicate that after immunization of mice with recombinant subunit vaccines NNR, TTR, and NTR, they can promote the activation of splenic T lymphocytes and lung TRM cells, thereby exerting immune protective effects.

### 3.4 Cross-protective efficacy of recombinant subunit vaccines NNR, TTR, and NTR in BALB/c mice and golden hamsters

Given the ability of tandem RBDs to produce potent broadly neutralizing antibodies and induce cellular immunity, we attempted to evaluate the efficacy of NNR, TTR, and NTR in protecting mice against heterologous SARS-CoV-2. To achieve this goal, the immunized BALB/c mice and golden hamsters were infected with SARS-CoV-2 BA.2 strain (Zhu et al., 2025; Kaur Sardarni et al., 2025).

### 3.5 NNR, TTR and, NTR protect BALB/c mice from challenges with live SARS-CoV-2

**Figure 7:**
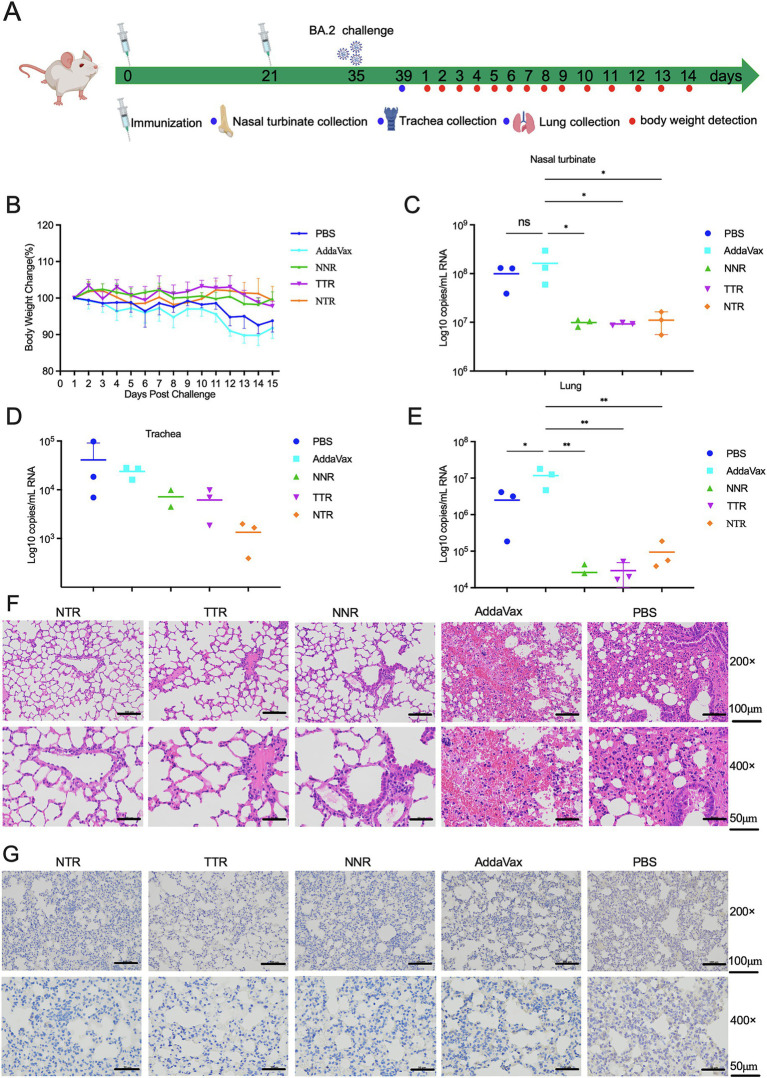
Evaluation of the protective efficacy of NNR, TTR, and NTR against SARS-CoV-2 BA.2 challenge in BALB/c mice. **(A)** Schematic illustration of the timeline of BALB/c mice immunization and challenge with SARS-CoV-2 BA.2; **(B)** Body weight changes after infection in each group; Fluorescent quantitative PCR was performed on the lung tissues **(C)**, nasal turbinate **(D)**, and trachea **(E)**. Histopathological examination of mice lung tissues, HE **(F)** and IC **(G)**, PBS and AddaVax as control. Scale bar, 100 μm (200×) and 50 μm (400×). *n* = 10 mice/group/time point. Data are shown as the means ± SD and were analyzed by one-way ANOVA (*p < 0.05, **p < 0.01, ***p < 0.001, ****p < 0.0001).

BALB/c mice were intramuscularly injected with three recombinant subunit vaccines at weeks 0 and 3, with control and adjuvant groups set up. Subsequently, at week 5, all BALB/c mice were transferred to a biosafety level 3 laboratory and challenged with SARS-CoV-2 BA.2 via intranasal inoculation (Figure 7A).

As expected, the control group BALB/c mice showed raised back hair, and anxiety on day 2 post-challenge. In contrast, the immunized groups of BALB/c mice showed no obvious clinical symptoms, smooth fur, normal eating and drinking, and no significant differences among the NNR, TTR, and NTR immunized groups. PBS- and adjuvant-treated mice may have slightly lost body weight compared to vaccinated mice, but this was not statistically significant (Figure 7B).

Next, on day 4 post-challenge, nasal turbinates, trachea, and lung tissues were collected from BALB/c mice, and real-time fluorescent quantitative PCR using primers targeting the ORF1a/b gene was performed to detect changes in viral load in various tissues of BALB/c mice in each group. The qPCR assay for ORF1ab exhibited an amplification efficiency of 98% and a limit of detection of 10 copies per reaction. Compared to the control group, the SARS-CoV-2 viral load in the organs of NNR, TTR, and NTR immunized groups decreased significantly. The greatest decrease in SARS-CoV-2 viral load was in lung tissue, followed by nasal turbinates, with the respiratory tract showing the least decrease. There were no significant differences among the NNR, TTR, and NTR immunized groups (Figures 7C–E).

Furthermore, H&E staining of lung sections showed that the control and adjuvant groups exhibited lung hemorrhage, vascular thrombosis, bronchial obstruction, and protein exudation, with increased inflammatory cells surrounding blood vessels and airways, accompanied by significant inflammatory cell infiltration. In contrast, the lung structure in immunized mice showed no obvious abnormalities, with only a small amount of lymphocyte infiltration visible around blood vessels (Figure 7F).

Further immunohistochemistry (IHC) staining of lung tissue sections using SARS-CoV-2 RBD antibody showed that the staining intensity in the lung tissue of immunized mice was lower than that of the control group, indicating that there was almost no SARS-CoV-2 BA.2 replication in the lung tissue of immunized mice, and lung injury was significantly reduced (Figure 7G).

### 3.6 NNR, TTR, and NTR protect golden hamsters from challenge with live SARS-CoV-2

**Figure 8:**
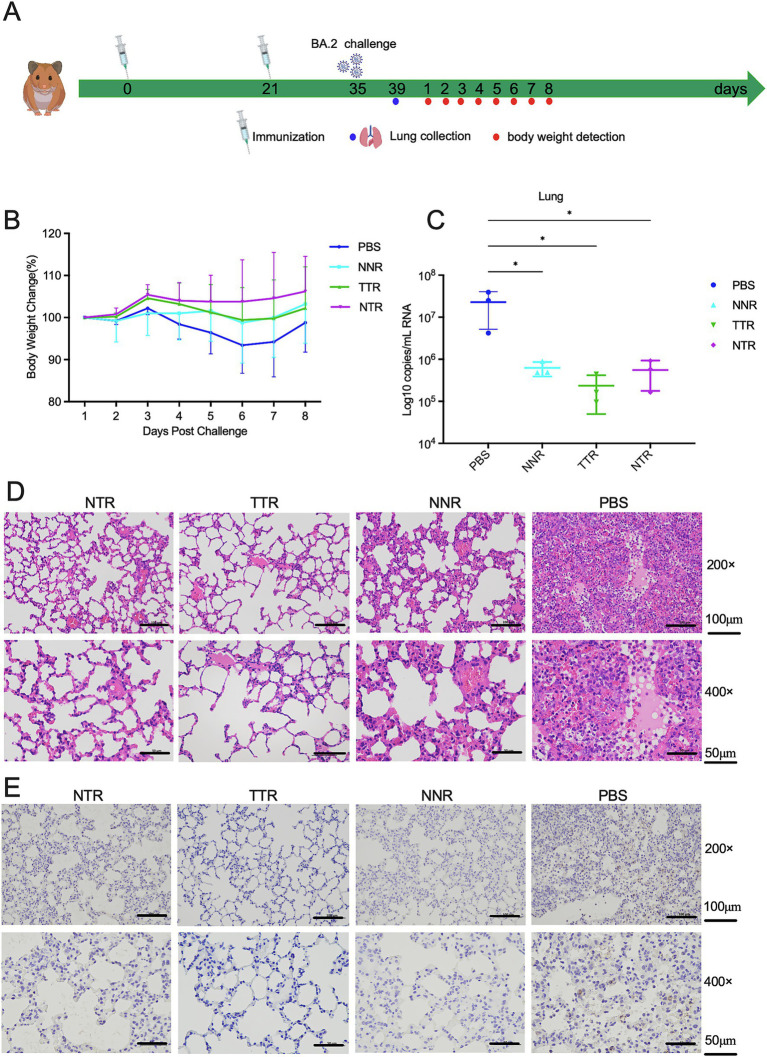
Evaluation of the protective efficacy of NNR, TTR, and NTR against SARS-CoV-2 BA.2 challenge in golden hamster. **(A)** Schematic illustration of the timeline of golden hamster immunization and challenge with SARS-CoV-2 BA.2; **(B)** Body weight changes after infection in each group; **(C)** Fluorescent quantitative PCR was performed on the lung tissues; Histopathological examination of mice lung tissues, HE **(D)** and IC **(E)**, PBS as control. Scale bar, 100 μm (200×) and 50 μm (400×). *n* = 8 mice/group/time point. Data are shown as the means ± SD and were analyzed by one-way ANOVA (*p < 0.05, **p < 0.01, ***p < 0.001, ****p < 0.0001).

To more realistically evaluate the protective effects of NNR, TTR, and NTR vaccines, we assessed the protective efficacy of three recombinant subunit vaccines against SARS-CoV-2 using golden hamsters. As in the previous procedure, golden hamsters were immunized twice, followed by a SARS-CoV-2 BA.2 challenge experiment 2 weeks later (Figure 8A).

On the second day after the challenge, the control group and adjuvant group golden hamsters showed a slow decrease in body weight, reduced food and water intake, ruffled fur, and clinical symptoms such as lethargy and sleepiness. In contrast, the NNR, TTR, and NTR immunized groups of golden hamsters showed no significant clinical symptoms, maintained stable body weights within the normal range of variation, and no significant differences were observed among the NNR, TTR, and NTR immunized groups. According to statistical analysis, there was no statistical significance in weight between the control and vaccinated groups (Figure 8B).

Subsequently, lung tissues were collected on the fourth day after the challenge, and changes in tissue viral load were monitored using the same method as before. Compared to the control group, there was a significant decrease in lung viral load, although NNR, TTR, and NTR did not show statistical differences among them (Figure 8C).

H&E staining of lung sections showed that in the control group, extensive alveolar wall thickening with infiltration of lymphocytes, neutrophils, and macrophages was observed, along with pulmonary edema and eosinophilic serous-like substances in the alveolar spaces. In the immunized groups, only mild thickening of alveolar walls with slight infiltration of lymphocytes and neutrophils was observed (Figure 8D).

Our further immunohistochemistry (IHC) staining experiments on lung tissue sections demonstrated that there was almost no SARS-CoV-2 BA.2 proliferation in the lung tissues of the immunized groups. NNR, TTR, and NTR provided protection to the lungs of golden hamsters during the viral challenge and inhibited viral replication in the lungs (Figure 8E).

## 4 Discussion

Since the beginning of the 21st century, human society has experienced three major coronavirus epidemics. The most recent occurrence was caused by the SARS-CoV-2 virus, which was first reported at the end of 2019 and rapidly evolved into the global COVID-19 pandemic (Lundstrom et al., 2023; Huang et al., 2021). During the 4 years of pandemic, the COVID-19 epidemic continued to have a far-reaching impact on the world. The emerging variant appears to lead to milder/less severe acute sequelae (also known as long COVID). Surprisingly, individuals who have experienced reinfection with these newly emerging variants are more likely to develop post-COVID-19 syndrome (Calvaresi et al., 2023; Wang et al., 2022). This syndrome can last for more than 3 months and is accompanied by the persistent presence of the virus in tissues throughout the body (Yang et al., 2020). As a highly variable RNA virus, SARS-CoV-2 exhibits significant transmissibility, pathogenicity, and immune evasion potential (Li et al., 2022; Pathirana and Kaparakis-Liaskos, 2016). Therefore, it is necessary to develop new vaccines with cross-immunity protective effects to control or prevent the COVID-19 pandemic.

The SARS-CoV-2 spike protein mediates virus entry into cells through its receptor-binding domain (RBD) (Dai and Gao, 2021; Dai et al., 2022; Wang et al., 2020). RBD is the main target of neutralizing antibodies (NAbs) and is an ideal antigen for vaccine development due to its immunodominance (Dai and Gao, 2021). The SARS-CoV-2 variant is the result of natural selection during continuous host transmission. The RBD structure contains multiple mutations located in a small 25 amino acid fragment at the top of the spike protein, mediating binding to the human ACE2 receptor and infecting humans. Cryo-electron microscopy (Cryo-EM) analysis shows that the RBD presents in a natural homotrimeric form (Wrapp et al., 2020; Kocharovskaya et al., 2025; Xu et al., 2021). Moreover, the N-terminus and C-terminus of the RBD sequence are structurally adjacent, allowing them to be connected end-to-end to form RBD trimers that mimic the natural arrangement of RBD. Additionally, the long flexible loops at both ends of the RBD can serve as natural linkers without the need for external sequences, maintaining the structural integrity of the various regions. The end-to-end connected RBD homotrimer has been reported to exhibit a regular shape and is assessed as a subunit vaccine with good immunogenicity (Liang et al., 2022; Wang et al., 2024). By analyzing the mutation sites in the RBD fragments of existing SARS-CoV-2 variants, we found that nearly all variants have mutations at five amino acid sites (417, 452, 478, 484, and 501), which have a certain impact on viral transmission and immune evasion. This strategy of constructing a single immunogen by hybridizing the RBD proteins from different circulating SARS-CoV-2 variants aids in the development of a multivalent vaccine with strong immunogenicity and good cross-immunity.

Currently, the approved recombinant protein vaccines based on RBD fragment design mainly include: Abdala vaccine (CIGB-66) based on RBD monomer, ZF2001 vaccine constructed with RBD dimer, and FINLAY-FR-2 (Soberana 02) vaccine based on multi-copy recombinant RBD. The efficacy of these vaccines has been confirmed in clinical trials. However, the effectiveness of existing COVID-19 vaccines against infection decreases over time, and their resistance to newly emerging variants also weakens (Yadav et al., 2023). To combat SARS-CoV-2 variants (Yang et al., 2021), it is suggested to use heterologous recombination strategies to construct RBD heterodimers and RBD heterotrimers (Zhang et al., 2022; Xu et al., 2022; Zhang et al., 2023). Compared to the homologous form of RBD, the heterologous multimeric RBD induces a broader neutralizing antibody spectrum and provides better protection against infections from various SARS-CoV-2 variants.

Based on this, we constructed a novel recombinant protein vaccine with cross-immunity protection, targeting key mutation sites in the RBD to address future mutating viruses. The five mutation sites K417N/T, L452R, E484K, and N501Y have well-documented roles in ACE2 binding, viral transmissibility, and immune escape across major SARS-CoV-2 variants of concern, including Alpha, Delta, and Omicron. These residues represent key immunodominant sites that are recurrently targeted by neutralizing antibodies, and their inclusion was intended to focus immune responses on functionally critical regions associated with broad protection (Han et al., 2022; Niu et al., 2024). Therefore, we constructed the new RBD proteins that contain all five mutation sites and used flexible peptides to link two RBDs in tandem, forming the novel recombinant coronavirus RBD proteins. Compared to the wild-type SARS-CoV-2 RBD protein, the mutated RBD will exhibit better adaptability to existing variants. Additionally, the tandem form of the RBD protein can significantly increase the neutralizing antibody titer and enhance the effectiveness of the vaccine compared to the single RBD protein (Qiao et al., 2022; Mambelli et al., 2023; Kumar et al., 2022). Theoretically, this protein vaccine has greater immunological advantages and will be able to more effectively induce the body’s immune response, compensating for the shortcomings of current vaccines and providing a new perspective for the development of future broad-spectrum COVID-19 vaccines.

To validate this idea, we conducted immunization and challenge experiments in mice and golden hamsters after successfully expressing high-purity recombinant proteins. Three recombinant subunit vaccines, NNR, TTR, and NTR, were used to immunize mice with adjuvants. The serum from the immunized group induced a stronger immune response after booster immunization and produced higher levels of neutralizing antibodies compared to the control group, generating a robust immune response. Notably, the recombinant protein in the TTR group showed higher antibody levels in specific IgG antibody and neutralizing antibody detection compared to other immunized groups, possibly due to the higher degree of affinity reduction in K417N compared to K417T (Barton et al., 2021; Xue et al., 2024). It is shown that further high-resolution epitope identification and structural dissection will be carried out in our follow-up study to clarify the detailed mechanism is necessary.

Strong specific T cell responses are usually accompanied by the secretion of various cytokines, which is crucial for effective prevention of viral infection. The detection results showed that CD4^+^ T cells and CD8^+^ T cells secreted high levels of cytokines IFN-γ, IL-2, and TNF-ɑ. From the perspective of cytokines, Th1 response is associated with IFN-γ secretion levels, while Th2 response is related to IL-4 secretion levels (Chen et al., 2012; Camiolo et al., 2021). Based on the higher titers of IgG1 compared to IgG2a in mouse immunized serum, and the greater amount of IL-4 than IFN-γ after splenocyte protein stimulation, although both IFN-γ and IL-4 increased to varying degrees, it is clear that the recombinant protein mainly favored a Th2 immune response after immunization. The observed Th2-biased response may enhance humoral immunity and antibody production, aiding viral neutralization, but could limit cross-protection against SARS-CoV-2 variants by reducing cross-reactive T-cell responses compared to Th1-dominated immunity. Additionally, Th2 skewing raises a theoretical risk of antibody-dependent enhancement (ADE), particularly if non-neutralizing antibodies are induced. However, the neutralizing antibodies generated in this study suggest low ADE risk, though formal evaluation in animal or *ex vivo* models is needed to confirm safety and fully assess the relationship between Th2 polarization, antibody breadth, and cross-protective efficacy. Notably, by comparing the results of the two proteins, the BA.2 RBD protein showed slightly lower stimulation levels than the original SARS-CoV-2 RBD protein, but the overall difference was not significant. This may be due to the vaccine immune escape effect caused by other amino acid mutations in Omicron (Ren et al., 2022), which also indicates the broad-spectrum protective activity of the recombinant protein after immunization in mice.

A suitable animal model should be able to produce viral infections with clinical symptoms similar to those observed in humans (DeGrace et al., 2022; Harnan et al., 2024). The golden hamsters which are susceptible, but infection self-resolves in most animals without mortality, mimicking moderate to severe (but mostly non-lethal), which is consistent with human infections (Qiao et al., 2022). In this study, two different animal models (BALB/c mice and golden hamsters) were used for challenge protection experiments. After intramuscular immunization, the weight changes of BALB/c mice and golden hamsters were continuously monitored, and they remained in a stable state compared to the control group, indicating that the animals were well protected. On the 4th day after the challenge, the virus titers in the lung tissues of BALB/c mice and golden hamsters were measured and showed a significant decrease compared to the control group. Similarly, HE staining and IHC results of lung tissue pathological sections showed reduced virus replication and significantly decreased pathological damage compared to the control group, with less lung injury observed in the lung tissue sections after immunization.

Overall, the three recombinant subunit vaccines can protect different animals after immunization and reduce viral infection of tissues. Our results also indicate that the RBD recombinant protein expressed through the baculovirus-insect cell expression system can induce specific immune responses and enhance protective antibody responses in animals, providing effective protection against SARS-CoV-2 challenge and inhibiting viral replication and release in mouse lungs. Although NNR, TTR, and NTR all reduced viral loads versus the control group, no significant differences were detected among them. These three dimeric RBD vaccines provided comparable protective efficacy against BA.2, likely because all elicited sufficient neutralizing antibodies to achieve near-maximal viral control, making further quantitative differences undetectable under our experimental model and detection range. Among the three recombinant subunit vaccines, the TTR recombinant protein has the best immune protective activity against the SARS-CoV-2 virus, significantly suppressing weight loss in animals and reducing viral replication in tissues and organs, exhibited the best immunogenicity. The TTR recombinant protein vaccine may be a safe and promising approach, providing preliminary data for the development of new COVID-19 vaccines with broad-spectrum protective activity.

It is worth noting that, as a preliminary exploration, this study has several limitations. First, a limitation of our mutation panel is that it was designed based on variants circulating through 2023 and does not fully represent the antigenic landscape of currently dominant variants such as JN.1 or its descendants. We acknowledge that broader coverage of additional emerging mutations may further improve cross-protection, and this will as a direction for future optimization. Second, it should be noted that all animal experiments were conducted using standard laboratory mouse strains rather than a humanized model such as hACE2-transgenic mice. Therefore, caution is warranted when extrapolating our findings to human infection conditions. Third, we have focused on evaluating immunogenicity and protective efficacy against the BA.2 variant as a representative strain to assess the potential cross-protection of our designed RBD construct, which includes mutations shared with several variants prior to early Omicron strains. Unfortunately, while our results partially support the potential of inducing cross-reactive immunity, we recognize that whether the observed immune protection extends to other emerging variants (e.g., BA.4/5, XBB, or JN.1) remains unknown. Further studies are needed to map the cross-neutralization profile against a broader panel of variants. Fourth, a key next step is to benchmark our platform against currently licensed vaccines under identical conditions.

In conclusion, we hope that countries around the world can unite, work together in research, and remain vigilant at all times to respond to future outbreaks of pandemics.

## Data Availability

The original contributions presented in the study are included in the article/Supplementary material, further inquiries can be directed to the corresponding authors.

## Footnotes

1
https://data.who.int/dashboards/covid19/cases?n=c
