# Correction: Recombinant RBD-based subunit vaccines incorporating high-frequency mutation sites elicit cross-immunity and robust protection against SARS-CoV-2

**DOI:** 10.3389/fmicb.2026.1925275

**Published:** 2026-07-22

**Authors:** Frontiers Production Office

**Affiliations:** Frontiers Media SA, Lausanne, Switzerland

**Keywords:** high-frequency SARS-CoV-2 RBD mutation sites, RBD, recombinant subunit vaccines, SARS-CoV-2, universal vaccine

Wrong affiliation, but not replacing with present address

Affiliation [^1^Henan Intelligent Diagnosis and Treatment Engineering Technology Research Center, The First Affiliated Hospital, Henan University, Kaifeng, China,

^2^Key Laboratory of Jilin Province for Zoonoses Prevention and Control, National Key Laboratory of Pathogen Microorganisms Biosafety, Academy of Military Medical Sciences, Changchun, China,

^3^State Key Laboratory of Antiviral Drugs, School of Pharmacy, School of Basic Medicine Sciences, Henan University, Kaifeng, China,

^4^Jiangsu Co-innovation Center for Prevention and Control of Important Animal Infectious Diseases and Zoonoses, Yangzhou University, Yangzhou, China,

^5^Kaifeng Key Laboratory for Infectious Diseases and Biosafety, The First Affiliated Hospital of Henan University, Kaifeng, China] was erroneously given as [^1^Henan Intelligent Diagnosis and Treatment Engineering Technology Research Center, The First Affiliated Hospital, School of Basic Medicine Sciences, Henan University, Kaifeng, China,

^2^State Key Laboratory of Antiviral Drugs, School of Pharmacy, Henan University, Kaifeng, China,

^3^Key Laboratory of Jilin Province for Zoonoses Prevention and Control, State Key Laboratory of Pathogen and Biosecurity, Academy of Military Medical Sciences, Changchun, China,

^4^Kaifeng Key Laboratory for Infectious Diseases and Biosafety, The First Affiliated Hospital of Henan University, Kaifeng, China,

^5^Jiangsu Co-innovation Center for Prevention and Control of Important Animal Infectious Diseases and Zoonoses, Yangzhou University, Yangzhou, China].

Changing author order

The order of authors in the author list of the published paper was erroneously given as:

[Zhiguang Ren^1, 2†^, Xiawei Liu^1, 3†^, Weiyang Sun^3†^, Xiaonan Gao^2^, Wei Jiang^1^, Yiwen Li^2^, Chengyu Hu^1^, Menghan Zhu^1^, Yongkun Zhao^3^, Yuanguo Li^3^, Xianzhu Xia^3^, Zhengyan Yang^1^, Jing Liu^1,2^^*^, Xinrui Lv^1,4^^*^, Tiecheng Wang^3,5^^*^ and Daxiang Cui^1^^*^]

The correct author list reads:

[Zhiguang Ren^1, 2†^, Xiawei Liu^1, 3†^, Weiyang Sun^3†^, Wei Jiang^1, 2^, Yiwen Li^1, 2^, Xiaonan Gao^2^, Chengyu Hu^1^, Menghan Zhu^1^, Yongkun Zhao^3^, Yuanguo Li^3^, Xianzhu Xia^3^, Zhengyan Yang^1^, Jing Liu^1,2^^*^, Xinrui Lv^1,5^^*^, Tiecheng Wang^3,4^^*^and Daxiang Cui^1^^*^]

The author contributions statement was erroneously given as [ZR: Methodology, Investigation, Writing – original draft. XLi: Formal analysis, Resources, Validation, Writing – original draft. WS: Software, Writing – original draft, Data curation. XG: Writing – original draft, Validation, Data curation. WJ: Software, Validation, Writing – original draft. YL: Software, Writing – original draft, Data curation. CH: Validation, Software, Data curation, Writing – original draft. MZ: Validation, Data curation, Writing – original draft. YZ: Methodology, Supervision, Writing – original draft. YuL: Formal analysis, Writing – original draft. XX: Supervision, Project administration, Writing – original draft. ZY: Methodology, Conceptualization, Writing – original draft. JL: Methodology, Writing – review & editing, Resources, Visualization. XLv: Project administration, Supervision, Writing – review & editing, Conceptualization. TW: Conceptualization, Methodology, Funding acquisition, Project administration, Writing – review & editing. DC: Methodology, Project administration, Conceptualization, Writing – review & editing.].

The correct author contributions statement is [ZR: Methodology, Investigation, Writing – original draft. XLi: Formal analysis, Resources, Validation, Writing – original draft. WS: Software, Writing – original draft, Data curation. WJ: Software, Validation, Writing – original draft. YiL: Software, Writing – original draft, Data curation. XG: Writing – original draft, Validation, Data curation. CH: Validation, Software, Data curation, Writing – original draft. MZ: Validation, Data curation, Writing – original draft. YZ: Methodology, Supervision, Writing – original draft. YuL: Formal analysis, Writing – original draft. XX: Supervision, Project administration, Writing – original draft. ZY: Methodology, Conceptualization, Writing – original draft. JL: Methodology, Writing – review & editing, Resources, Visualization. XLv: Project administration, Supervision, Writing – review & editing, Conceptualization. TW: Conceptualization, Methodology, Funding acquisition, Project administration, Writing – review & editing. DC: Methodology, Project administration, Conceptualization, Writing – review & editing.].

The Ethics statement was erroneously given as [The animal study was approved by the Animal Welfare and Ethics Committee of Changchun Veterinary Research Institute, Chinese Academy of Agricultural Sciences. The study was conducted in accordance with the local legislation and institutional requirements.].

The correct Ethics statement is [The animal study was approved by the Animal Welfare and Ethics Committee of Henan University, and Key Laboratory Jilin Province for Zoonoses Prevention and Control, National Key Laboratory of Pathogen Microorganisms Biosafety, Academy of Military Medical Sciences. The study was conducted in accordance with the local legislation and institutional requirements.].

Adding/removing text

[Redundant institutional information of the animal ethics review committee was present in *Subsection 2.1 Facilities and ethics statement*].

A correction has been made to the section [**2 Materials and methods**, **subsection 2.1 Facilities and ethics statement**, Paragraph 1]:

“[All Animals were treated in accordance with Chinese welfare and ethical guidelines for laboratory animals. The protocol was approved by the Animal Welfare and Ethics Committee. All highly pathogenic virus experiments were performed in a Biosafety Level 3 (BSL3) facility.]”

Adding/removing text

[The laboratory name corresponding to Affiliation 2 was written incorrectly in *Subsection 2.2 Viruses, cells, antibodies, and animals*].

A correction has been made to the section [2 **Materials and methods**, *subsection 2.2 Viruses, cells, antibodies, and animals*, Paragraph 1]:

“[SARS-CoV-2 (IME-BJ05-2020, GenBank Accession No. MT291835) and SARS-CoV-2 BA.2 (hCoV-19/ Jilin/JSY-CC5/2022, GISAID Accession No. EPI_ISL_18435548) are kept in the laboratory of Key Laboratory of Jilin Province for Zoonoses Prevention and Control, National Key Laboratory of Pathogen Microorganisms Biosafety, Academy of Military Medical Sciences. Insect baculovirus system dual expression vector pFastBac1, Spodoptera frugiperda (Sf9) cells, insect cell High Five suspension cells, and Vero E6 cells were prepared and preserved in the laboratory of Key Laboratory of Jilin Province for Zoonoses Prevention and Control, National Key Laboratory of Pathogen Microorganisms Biosafety, Academy of Military Medical Sciences. Rabbit anti-SARS-CoV-2 (COVID-19) Spike RBD antibody (GTX135385) was purchased from GeneTex, Goat Anti-Rabbit IgG H&L (FITC) (ab6717) and HRP-conjugated Goat Anti-Rabbit IgG (ab6721) were purchased from Abcam. Young female BALB/c mice aged 6–8 weeks, and elderly female BALB/c mice aged 6–8 months and female golden gophers aged 4–6 weeks were purchased from Beijing Viton Lever Laboratory Animal Technology Co. The animals were kept under specific pathogen-free conditions, with ad libitum access to food and water. Before immunization or virus challenge, animals are acclimated to the environment for 7 days, and monitored twice daily.]”

The original version of this article has been updated.

