# Supplementary figures and images for: Recombinant RBD-based subunit vaccines incorporating high-frequency mutation sites elicit cross-immunity and robust protection against SARS-CoV-2
Source: Front Microbiol. 2026 Jun 9;17:1806270. doi: 10.3389/fmicb.2026.1806270 (PMC13286944; doi:10.3389/fmicb.2026.1806270)

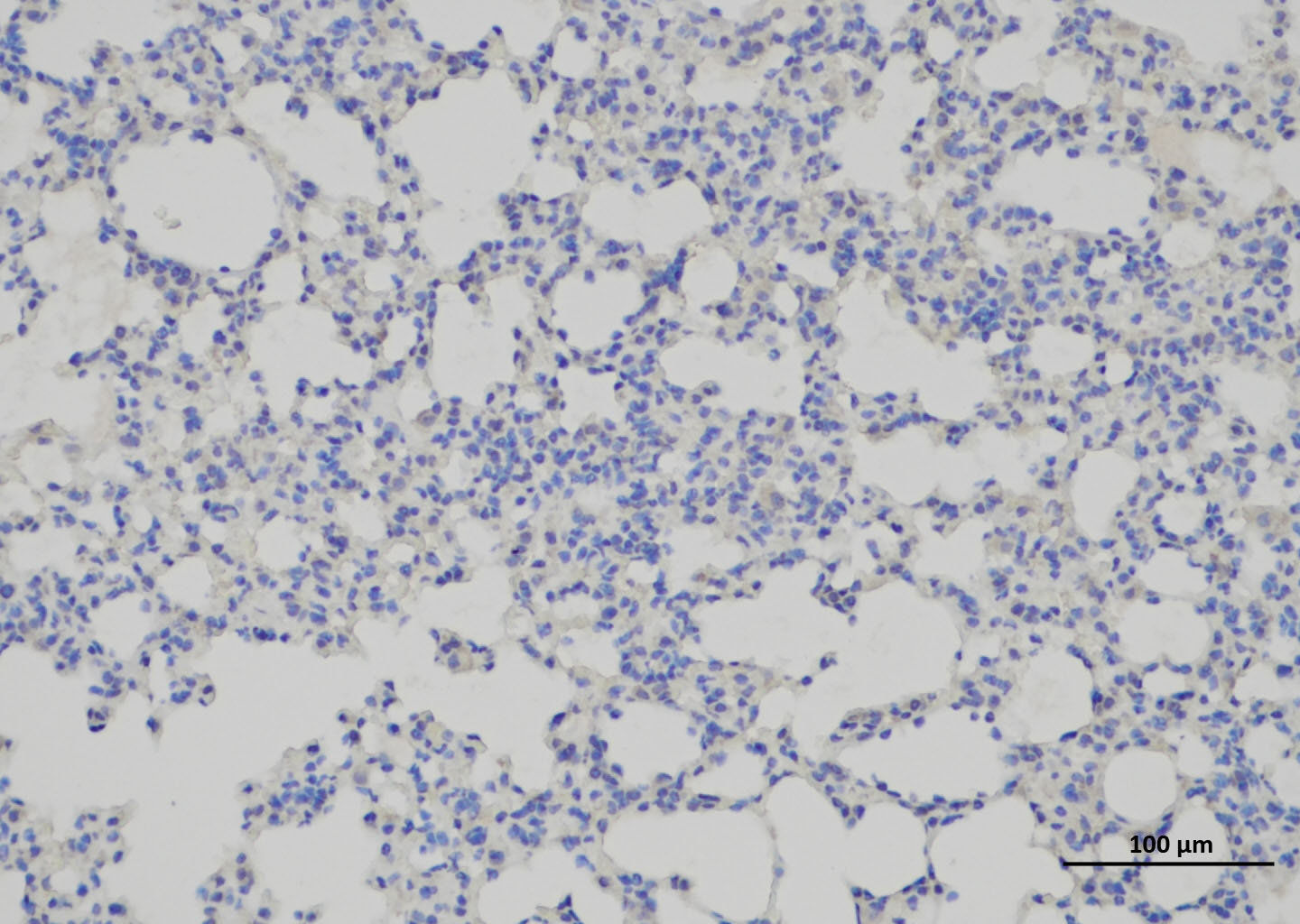

Supplement: Supplementary file 1 [file Image_1.JPEG]

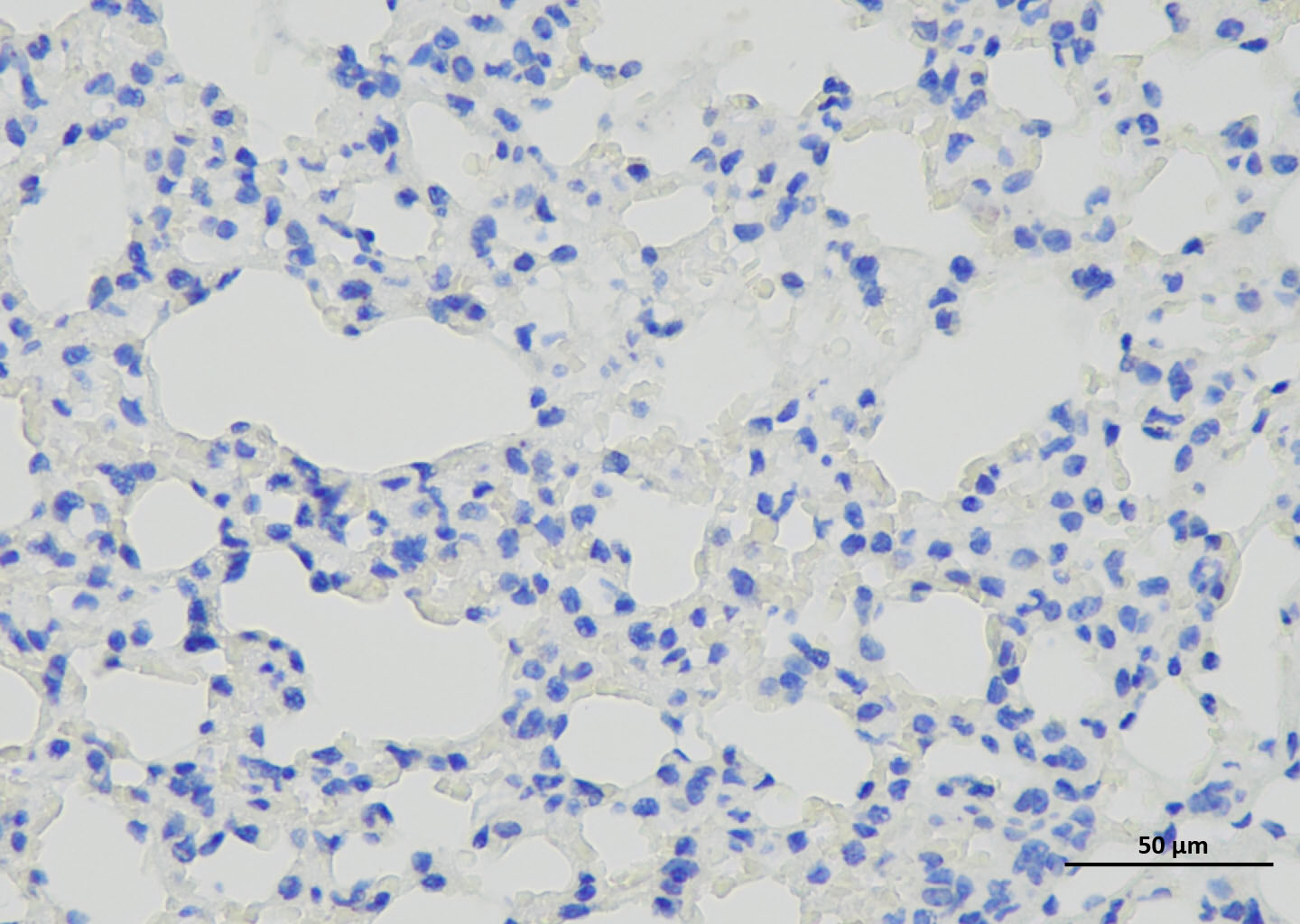

Supplement: Supplementary file 2 [file Image_10.JPEG]

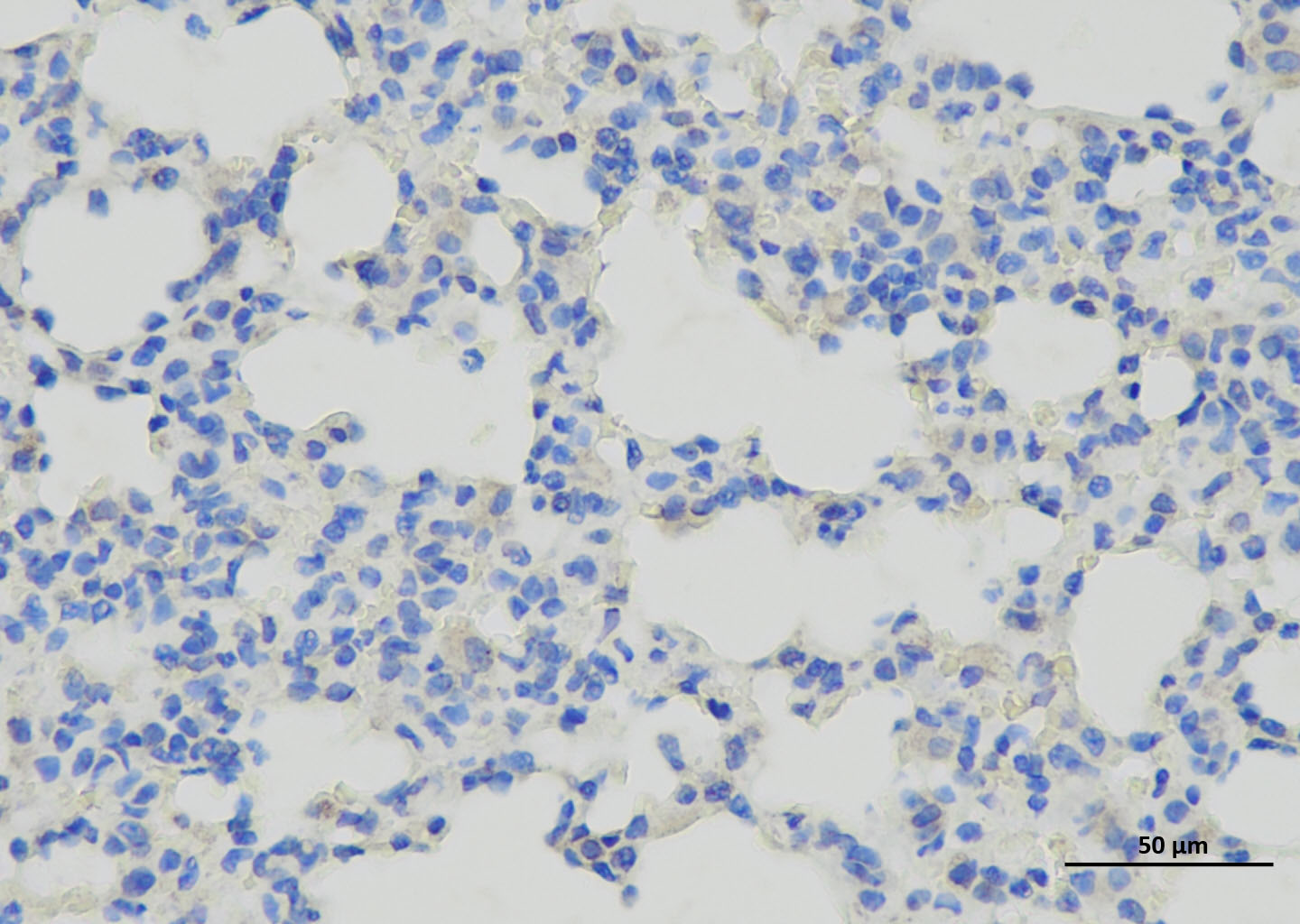

Supplement: Supplementary file 3 [file Image_2.JPEG]

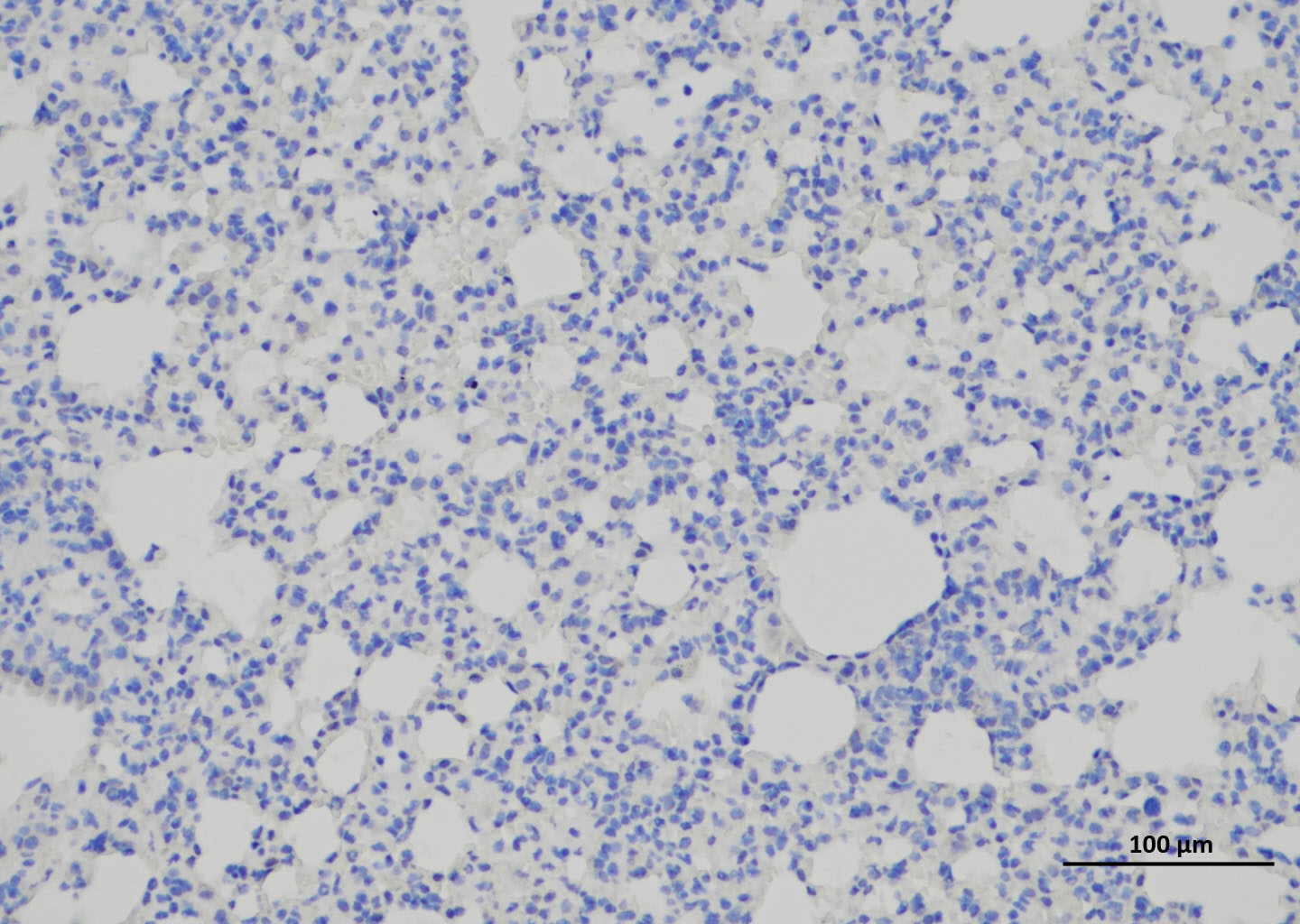

Supplement: Supplementary file 4 [file Image_3.JPEG]

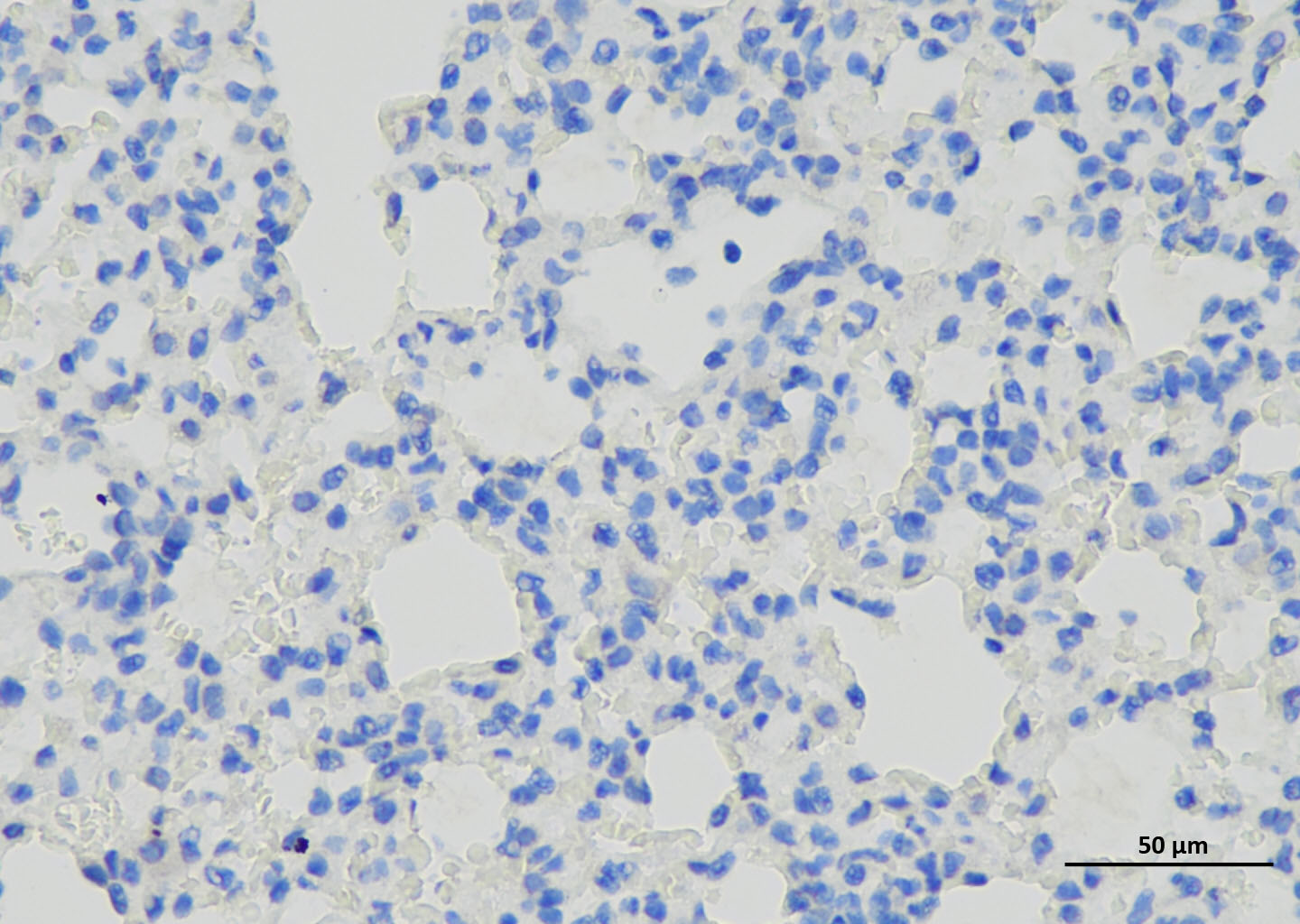

Supplement: Supplementary file 5 [file Image_4.JPEG]

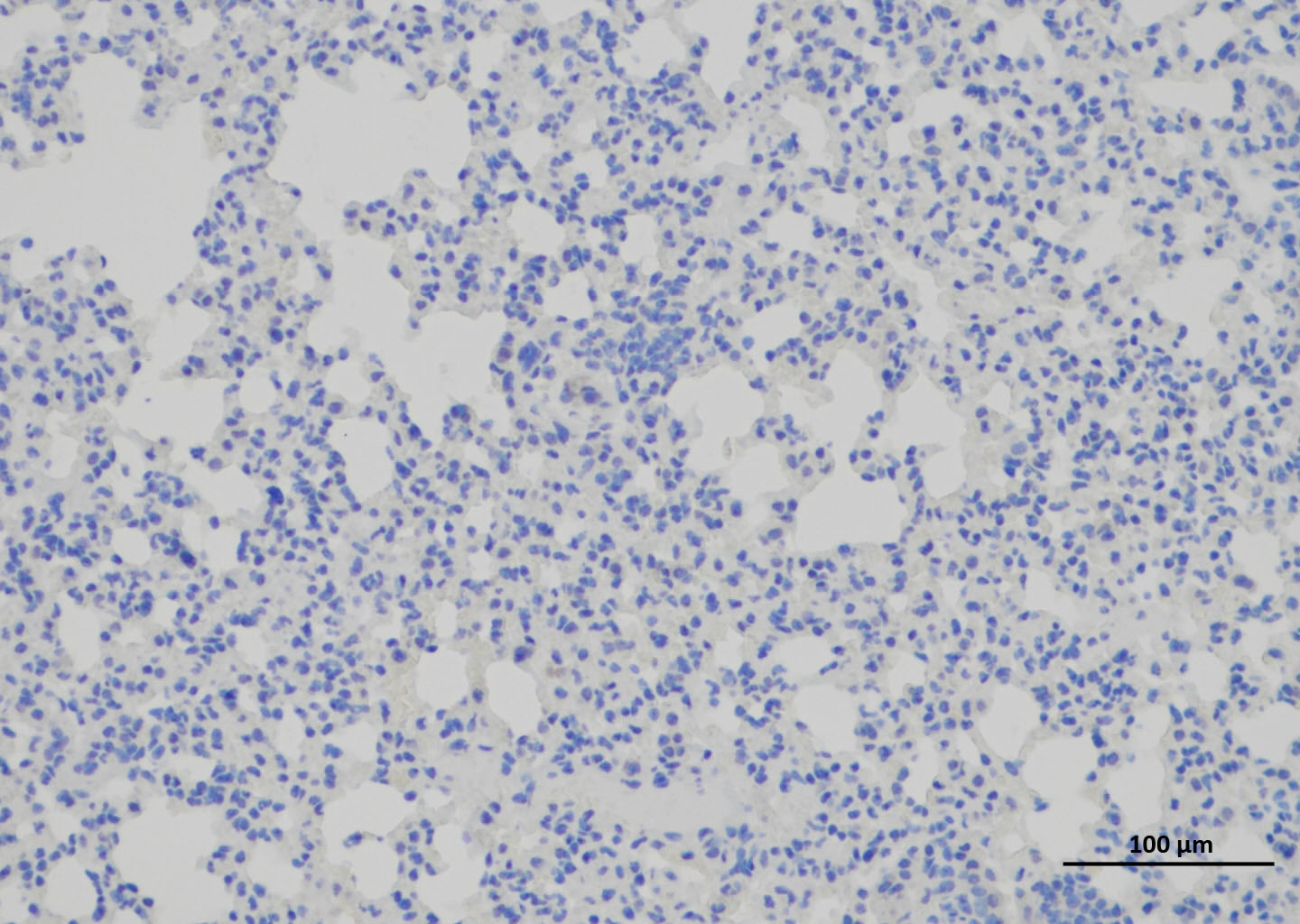

Supplement: Supplementary file 6 [file Image_5.JPEG]

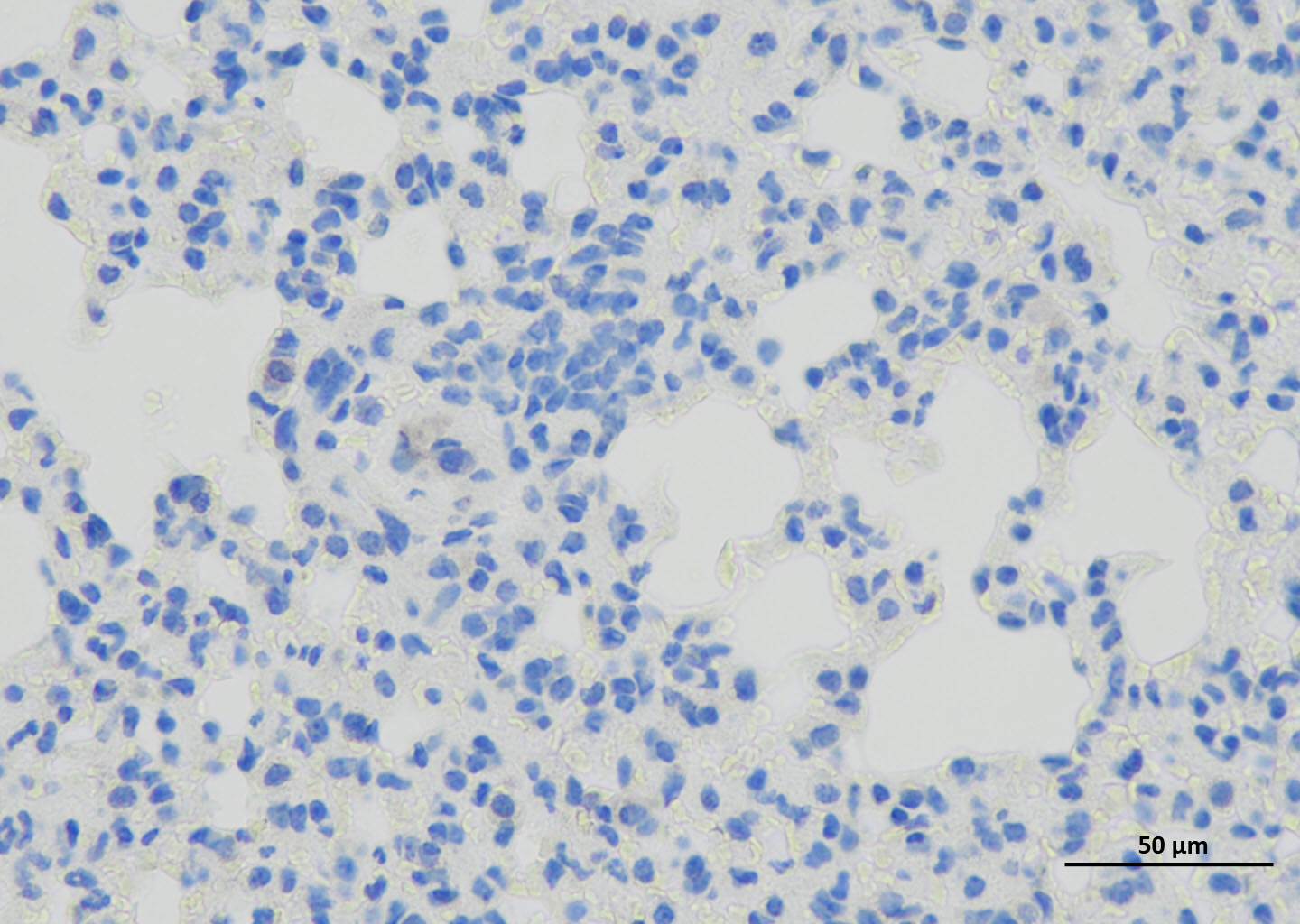

Supplement: Supplementary file 7 [file Image_6.JPEG]

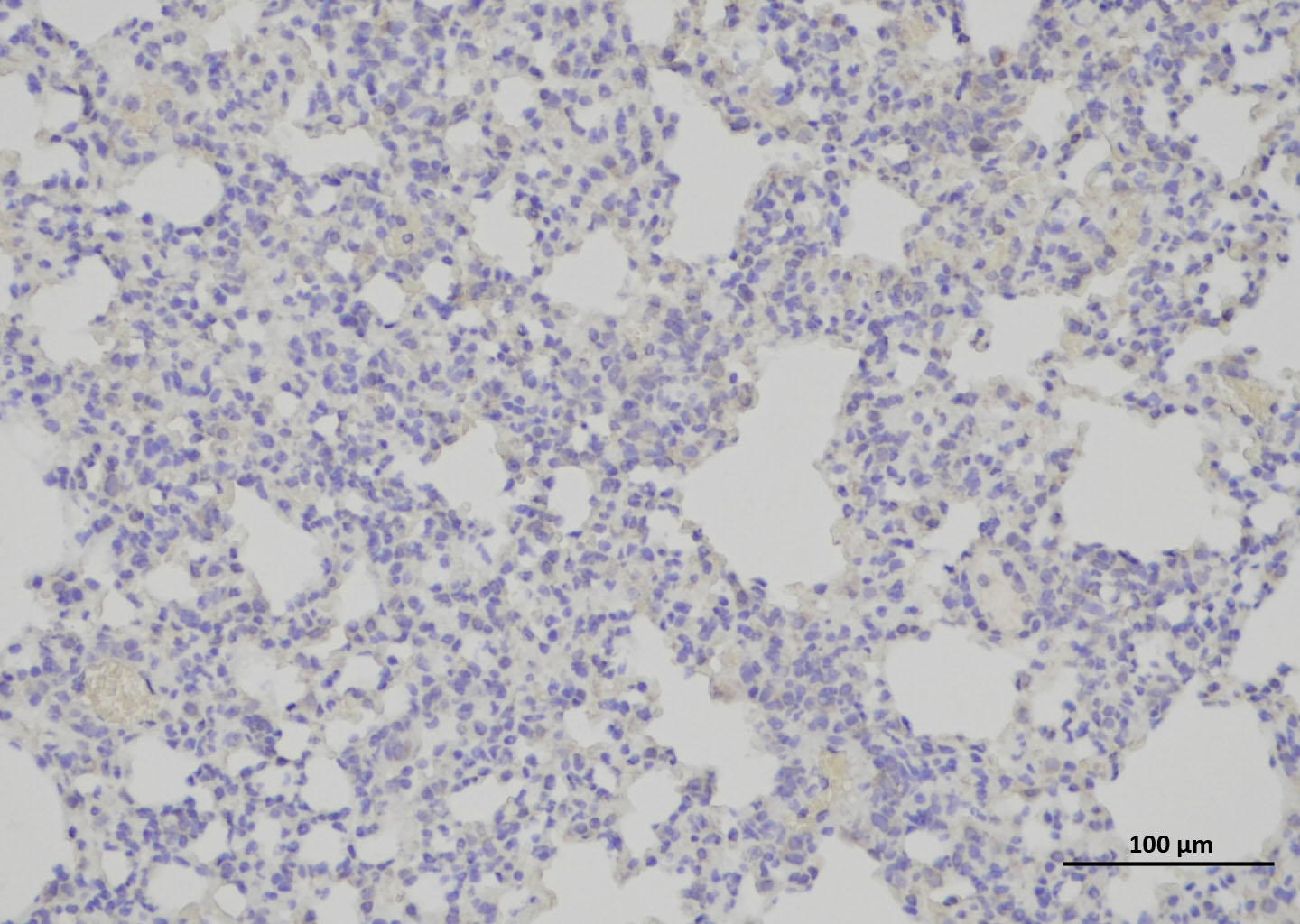

Supplement: Supplementary file 8 [file Image_7.JPEG]

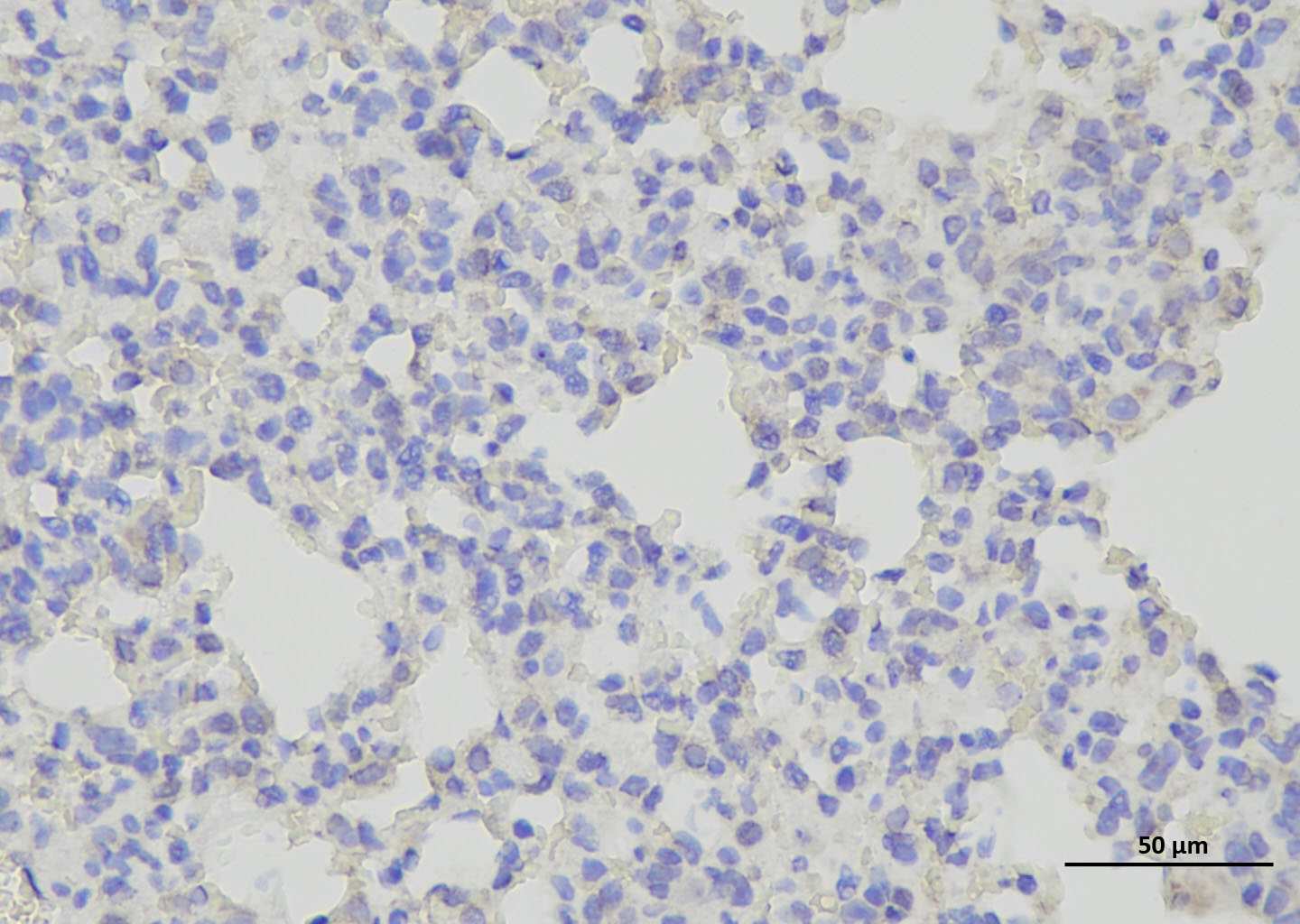

Supplement: Supplementary file 9 [file Image_8.JPEG]

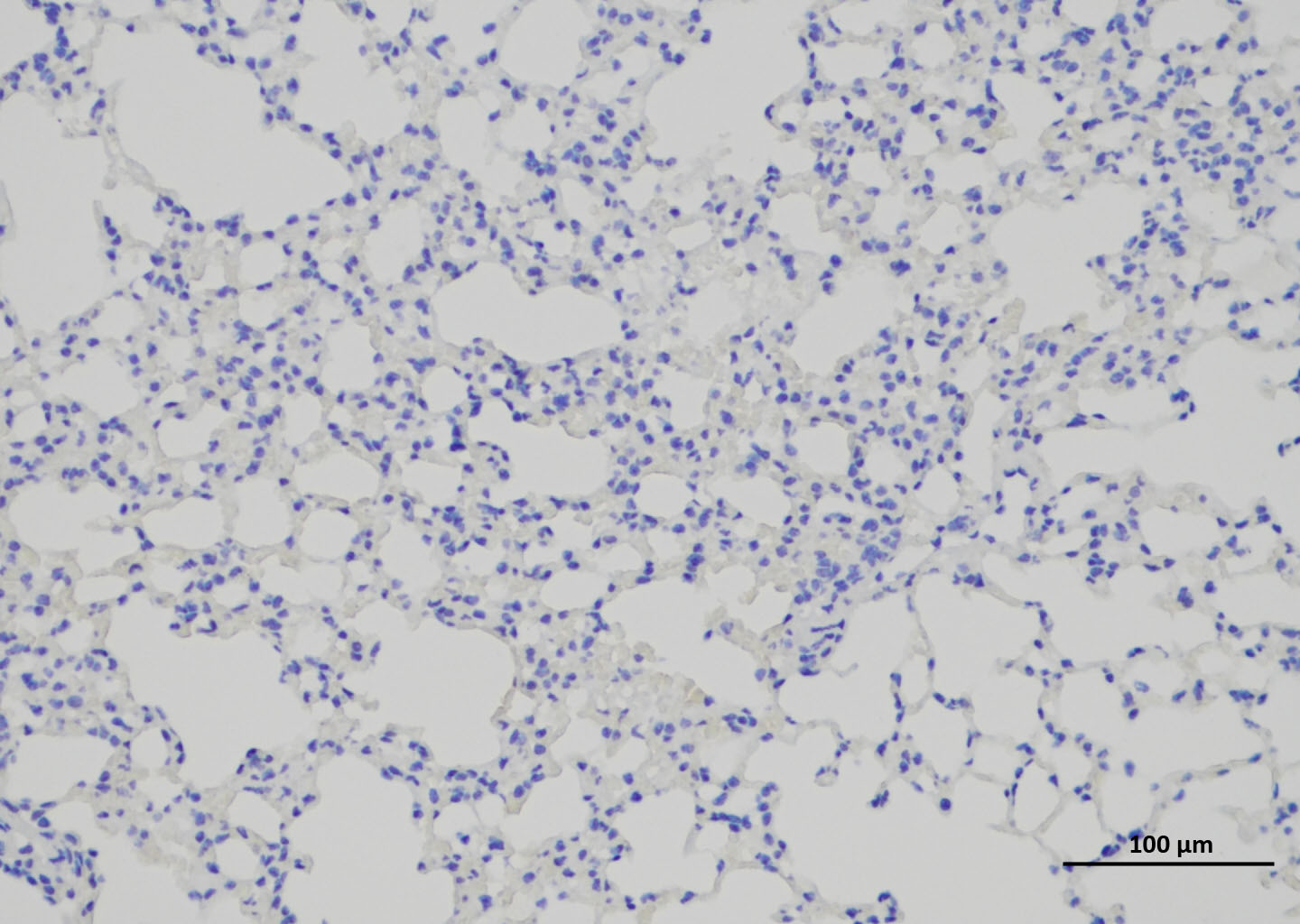

Supplement: Supplementary file 10 [file Image_9.JPEG]
